# Molecular affinity rulers: systematic evaluation of DNA aptamers for their applicabilities in ELISA

**DOI:** 10.1093/nar/gkz688

**Published:** 2019-08-08

**Authors:** Michiko Kimoto, Yun Wei Shermane Lim, Ichiro Hirao

**Affiliations:** 1 Institute of Bioengineering and Nanotechnology, 31 Biopolis Way, The Nanos, #07-01, Singapore 138669, Singapore; 2 NUS High School of Mathematics and Science, 20 Clementi Avenue 1, Singapore 129957, Singapore

## Abstract

Many nucleic acid aptamers that bind to target molecules have been reported as antibody alternatives. However, while the affinities of aptamers vary widely, little is known about the relationship between the affinities and their applicabilities for practical use. Here, we developed molecular affinity rulers: a series of DNA aptamers with different affinities that bind to the same area of target molecules, to measure the aptamer and its device applicabilities. For the ruler preparation, we used high-affinity DNA aptamers containing a hydrophobic unnatural base (Ds) as the fifth base. By replacing Ds bases with A bases in Ds-DNA aptamers targeting VEGF_165_ and interferon-γ, we prepared two sets of DNA aptamers with dissociation constants (*K*_D_) ranging from 10^−12^ to 10^−8^ M. Using these molecular affinity rulers, we evaluated the sensitivity of DNA aptamers in ELISA (enzyme-linked immunosorbent assay), which showed the clear relationship between aptamer affinities and their detection sensitivities. In sandwich-type ELISA using combinations of aptamers and antibodies, aptamers with *K*_D_ values lower than ∼10^−9^ M were required for sufficient sensitivities (limit of detection (LOD) < 10 pM) and signal intensities, but optimizations improved the lower-affinity aptamers’ applicabilities. These aptamer affinity rulers could be useful for evaluating and improving aptamer applicabilities.

## INTRODUCTION

Since 2015, >1000 reports on the generation and application of nucleic acid aptamers that bind to target molecules have been published each year. There are many modified methods for aptamer generation based on the evolutionary engineering method called SELEX (systematic evolution of ligands by exponential enrichment) (1,2) using DNA, RNA or modified nucleic acid libraries (3–12). Nevertheless, only a few types of aptamers have practical uses including modified DNA aptamers, such as SOMAmers (13–15) for diagnostics, and the modified RNA aptamer pegaptanib (Macugen) (16–18) that binds to vascular endothelial cell growth factor-165 (VEGF_165_) for the treatment of neovascular age-related macular degeneration, although several aptamers are currently in clinical trials (19–21). The affinities of the aptamers generated so far vary widely, and the *K*_D_ values of typical nucleic acid aptamers that bind to proteins range from 10^−12^ to 10^−6^ M. However, until now, there have been no reports that systematically examined the correlation between aptamer affinities and their applicabilities for practical use. To this end, a series of aptamers with different affinities that bind to the same area (epitope) of target molecules in similar binding modes is required as molecular affinity rulers. However, it is not easy to prepare such aptamer sets using conventional aptamer technologies, because of their limited affinities.

**Figure 1.**
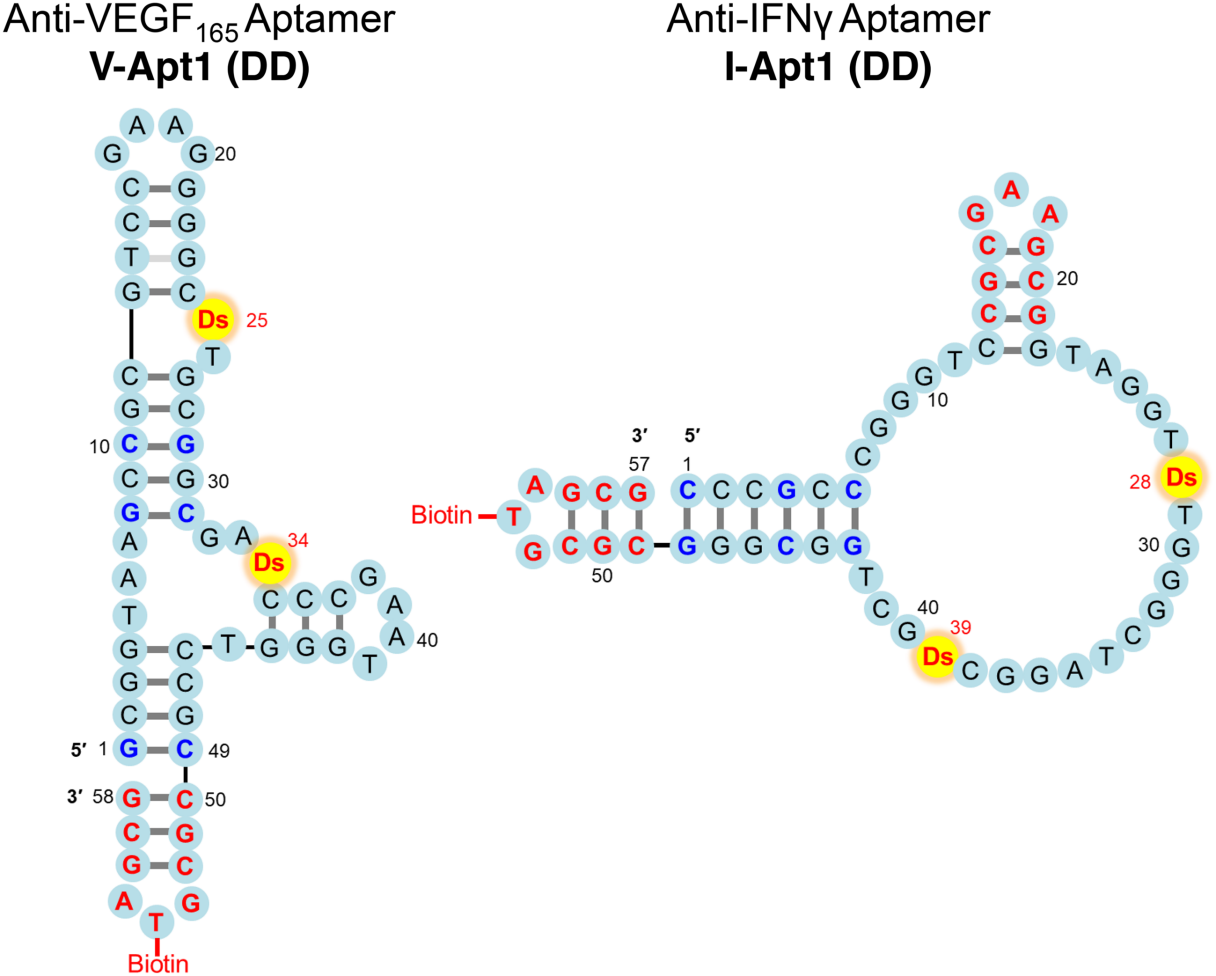
Presumed secondary structures of optimized anti-VEGF_165_ and anti-IFNγ UB-DNA aptamers containing two Ds bases. The G–C pairs shown in blue are changed from the A–T pairs or newly added in each original aptamer selected by ExSELEX, to strengthen the stability of the aptamer stem region. Mini-hairpin DNA sequences (CGCGAAGCG or CGCGTAGCG) shown in red also contribute to enhance the thermal stability of each aptamer, without any loss of binding affinity to the target. The thymidines within the mini-hairpin DNA sequences are used as the biotinylation sites.

Previously, we developed a new method to generate high-affinity DNA aptamers by introducing an artificial fifth base (unnatural base), using genetic alphabet expansion for SELEX (ExSELEX) (22). Adding just a few (one or two) hydrophobic unnatural bases (7-(2-thienyl)imidazo[4,5-*b*]pyridine, Ds) into DNA aptamers significantly augments their affinities to target proteins and cells (22–24). The unnatural-base DNA (UB-DNA) aptamers targeting vascular endothelial cell growth factor-165 (VEGF_165_) and interferon-γ (IFNγ) contain two essential Ds bases, and the *K*_D_ values of the optimized versions are around 1 and 33 pM, respectively (25,26). The optimization process includes stabilization by replacing A–T pairs with G–C in stem regions and by introducing an extraordinarily stable mini-hairpin DNA sequence (27,28) at the 3′-termini and/or internal stem-loops (Figure 1) (10,25,26). Since the natural-base variants of these aptamers obtained by replacing the Ds bases with A bases exhibit reduced affinity, we can prepare a series of DNA aptamers with different affinities that bind to the same epitopes on target proteins, by replacing either one or two Ds bases with A bases. Here, we report the preparation of aptamer affinity rulers: two sets of DNA aptamers with various affinities (10^−12^ to 10^−8^ M) and different binding modes targeting VEGF_165_ and IFNγ. Although we still lack sufficient tertiary structure information for both aptamer−target complexes, the previous data revealed that the anti-IFNγ DNA aptamer involving the G-quartet that binds to one IFNγ molecule is univalent, and the anti-VEGF_165_ DNA aptamer that binds to one VEGF_165_ -dimer molecule is bivalent. Using these molecular affinity rulers, we evaluated the application of these aptamers to an enzyme-linked immunosorbent assay (ELISA) (29).

ELISA is a fundamental and popular method for the quantitative detection of a wide range of targets with high sensitivity, with the use of antibodies. Its applications are not only limited to biochemical research but also extended to medicine, industrial quality-control testing and clinical diagnostics (30). In sandwich-type ELISA, a pair of antibodies is used: one (capture agent) is immobilized on a well to capture target epitopes and the other (detector agent) binds to different areas on the target for detection by coloration. The relationship between the antibody affinities and the target-detection sensitivities in ELISA has been reported (31–33). For example, the affinities of four anti-ABX10 antibodies (*K*_D_ = 0.057, 7.2, 30, 340 nM) correlated well with their sensitivities (LOD = 17, 26 000, 344 000 and 792 000 ng/ml, respectively) in ELISA as the bridging detector agents (31).

Besides antibodies, nucleic acid aptamers can also be used for ELISA. The first application of an anti-VEGF_165_ modified RNA aptamer (a prototype of Macugen) (34) in an ELISA format was reported in 1996, as an enzyme-linked oligonucleotide assay (ELONA) (35). Many aptamers have been tested and reported as alternatives to antibodies in ELISA and other biosensor formats (21,36–41). However, the practical widespread use of aptamers in diagnostic areas is still limited, and no systematic characterization and validation of aptamers, especially regarding the affinities required for the sensitive detection of their targets, have been reported.

Using our aptamer sets with various affinities, we examined their target detection sensitivities and signal intensities via ELISA (or ELONA or enzyme-linked aptamer assay (ELAA)). A combination of aptamers and antibodies was employed as the capture and detector agents in the ELISA experiments. The data revealed that the affinities of the aptamers correlated well with their ELISA sensitivities, and aptamers with higher affinities exhibited better sensitivities and signal intensities. In ELISA with aptamer–antibody sandwich systems, DNA aptamers with *K*_D_ values lower than ∼10^−9^ M were required for the high sensitivities (LOD < 10 pM) and sufficient signal intensities. Furthermore, using the molecular affinity rulers in ELISA, we found that the optimal concentration of the UB-DNA aptamers when used as capture agents was much lower than that of the non-UB-DNA aptamers: higher concentrations of the UB-DNA aptamer significantly reduced the signal intensities of the outputs, while their LODs were similar to those at the lower concentrations. By improving the immobilization method of the DNA aptamers, the signal intensity of the output even at the high concentration was greatly improved, not only for the UB-DNA aptamers but also for the non-UB-DNA aptamers. The data confirmed that the DNA aptamer sets could be useful as molecular affinity rulers to evaluate the aptamers’ potential and to improve the methods and systems for diagnostic and therapeutic applications, as the first part of the screening and optimization process.

## MATERIALS AND METHODS

### Reagents

The DNA fragments used in this study, listed in Supplementary Table S1, were chemically synthesized with an H8 DNA/RNA Synthesizer (K&A Laborgeraete), by using natural-base and biotin-dT phosphoramidites from Glen Research and a Ds phosphoramidite synthesized in-house, as described previously (42). The chemically synthesized DNAs were purified by denaturing gel electrophoresis. The mouse monoclonal anti-VEGF antibodies (clones 26503, VG76e, 16F1 and VG1) and anti-IFNγ antibodies (clones B133.5 and 2G1) were obtained from Thermo Fisher Scientific. Rabbit anti-biotin IgG (A150-109A) was purchased from Bethyl Laboratories, Inc. Recombinant human VEGF_165_ and IFNγ were obtained from PeproTech. The streptavidin-HRP conjugate (1 mg/ml) and the anti-mouse IgG HRP conjugate (1 mg/ml) were obtained from Jackson ImmunoResearch and Promega, respectively. Stock solutions (10×) for PBS and D-PBS(–) were obtained from Thermo Fisher Scientific and Nacalai Tesque, respectively. BSA, streptavidin, and Tween 20 were purchased from Promega. Nonidet P-40 was purchased from Nacalai Tesque. The TMB-substrate solution was purchased from KPL. Human serum (lot # SLBS8635) was obtained from Sigma-Aldrich.

### Biotinylation of antibodies

For biotinylation, each antibody solution (1 μM in 1× PBS) was mixed with Thermo Scientific™ EZ-Link™ Sulfo-NHS-LC-Biotin (final concentration of 45 μM), and the mixture was incubated at room temperature for 20 min. The antibody was then recovered after desalting, using Amicon Ultra-0.5 Centrifugal Filter Units (MWCO: 50 000). The biotinylated antibody solutions in 1× PBS were kept at 4°C until use.

### Surface plasmon resonance (SPR) analysis

Binding affinity profiles were obtained at 25°C on a Biacore T200 (GE Healthcare) using running buffer composed of 1× PBS supplemented with 0.05% Nonidet P-40 (and additional 50 mM NaCl for anti-IFNγ aptamer analysis (25)). For the immobilization of each ligand (aptamer variant or monoclonal antibody), we used streptavidin-coated sensor chips and immobilized biotinylated molecules on the flow cell, by injecting 0.5 nM of the ligand solution in running buffer at a flow rate of 0.5 μl/min. The injection times for immobilization were 60 sec for the anti-VEGF_165_ DNA aptamer variants, 480 sec for the anti-IFNγ DNA aptamer variants, 960 sec for B133.5 and 2G1 (mouse monoclonal anti-IFNγ antibodies), and 120 sec for 20653 (mouse monoclonal anti-VEGF_165_ antibody). Binding kinetic profiles were monitored by injecting at least five different concentrations of the analyte solutions (VEGF_165_ or IFNγ, 0.156–40 nM) for 150 sec (binding), at a flow rate of 100 μl/min. The analyte dissociation patterns were then recorded for 450 sec. To regenerate the ligand on the flow cell surface, denaturation solutions (50 mM NaOH for aptamers and 10 mM glycine, pH 2.5, for antibodies) were injected for 5 sec, and then the ligand was equilibrated in running buffer for 10 min. The kinetic parameters for the target binding, association rates (*k*_on_), dissociation rates (*k*_off_), and dissociation constants (*K*_D_ = *k*_off_/*k*_on_), were determined with the BIAevaluation software version 3.0, by using the double-reference subtraction method and global curve fitting (more than twice at each concentration) to a 1:1 Langmuir model.

### Gel-mobility shift assay

The aptamer–protein complex formation was analyzed by gel electrophoresis. Each DNA aptamer variant (25 nM final concentration) was mixed with each target protein (50 nM final concentration) in 1× PBS supplemented with 0.05% Nonidet P-40 (20 μl solution). After an incubation at 25°C for 30 min, 5 μl of 25% glycerol was added and 6 μl portions of the solutions were immediately subjected to PAGE (8% polyacrylamide gel containing 5% glycerol in 0.5× TBE, with or without 3 M urea) at ∼30°C. The band patterns on the gels, stained with SYBR Gold, were detected with a bioimaging analyzer, LAS-4000 (Fuji Film).

### Preparation of 96-well plates to immobilize/capture targets

For the direct immobilization of the target, microtiter plates (MaxiSorp™ 96-well plates from Nunc) were coated for 2 h at room temperature with 100 μl/well of 0, 1.25, 5 or 20 nM VEGF_165_ or IFNγ solution, diluted with 1 μg/ml BSA in 0.1 M sodium carbonate buffer (pH 9.6). For the sandwich-type target immobilization, the plates were first coated with 50 μl/well of a 5 μg/ml solution of streptavidin or anti-biotin IgG (for use of aptamers as capture agents), or a 3 μg/ml solution of the respective monoclonal antibody (for use of aptamers as primary detector agents) in the carbonate buffer. The plates were blocked by filling the wells with 300 μl of 10 mg/ml (1%) BSA in 1× D-PBS(–) for at least 2 h at room temperature. When using aptamers as capture agents, streptavidin-coated wells were washed once with 200 μl of wash buffer (1× D-PBS(–) with 0.05% Tween 20), and then incubated with 100 μl of aptamer variant solution (1–150 nM) in binding buffer (1× D-PBS(–) with 0.05% Tween 20 and 0.1% BSA) at room temperature for 30 min. The aptamer or antibody-coated wells were washed three times with 200 μl of washing buffer. The wells were then incubated with 100 μl of target solutions (10, 50 or 250 pM, or serial 2-fold dilutions to determine the limit of detection (LOD)) in binding buffer for 30 min at room temperature, followed by one wash with 200 μl of washing buffer.

### ELISA/ELONA detection process

To each well, 100 μl of 10 nM of primary detector solution (aptamer, non-biotinylated monoclonal antibody or biotinylated monoclonal antibody) in binding buffer was added and incubated for 30 min (for sandwich-type target immobilization) or for 60 min (for direct target immobilization). After washing the wells once, 100 μl of secondary detector solution (Streptavidin–HRP conjugate for biotinylated primary detector or anti-mouse IgG HRP conjugate, diluted to 1:20 000 with binding buffer) was added to each well, and then incubated for 30 min. After washing the wells six times, 100 μl/well of TMB-substrate solution was added and incubated for 15 min, unless otherwise indicated. After adding 100 μl of 0.1 M HCl to the well to stop the reaction, the absorbance of the wells at 450 nm (OD_450_) was measured on a multimode plate reader (TECAN INFINITE 2000 or BioTek Cytation 3). The assays under each condition were performed at least in duplicate (*n* ≥ 2), and the average absorbance data (combined from several independently repeated experiments) are shown in the graphs with error bars, which represent one standard deviation.

## RESULTS

### Preparation of molecular affinity rulers: a series of DNA aptamer sets with different *K*_D_ values to their same targets

**Figure 2.**
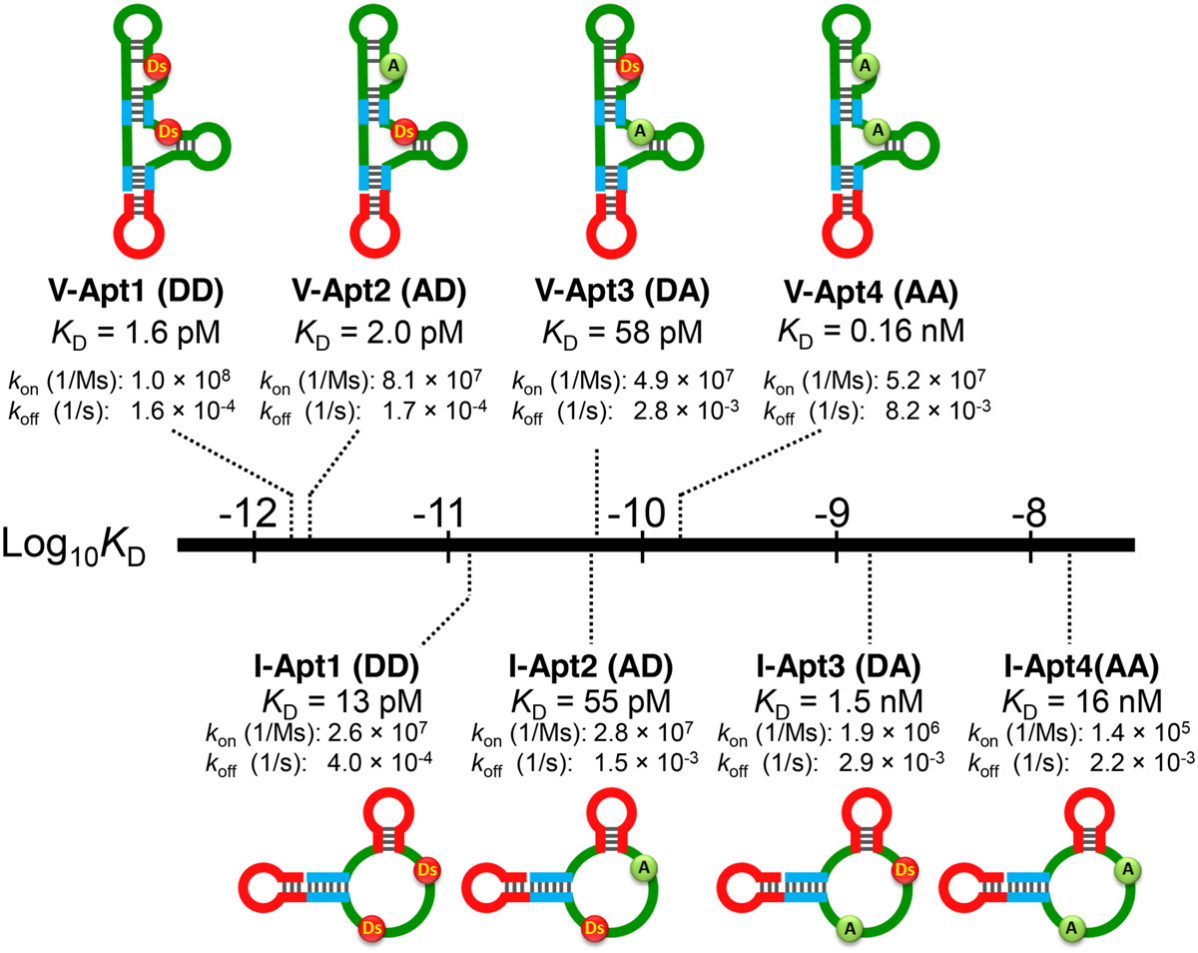
Molecular affinity rulers: distribution of the target affinities of the anti-VEGF_165_ and anti-IFNγ aptamer variants. By replacing Ds with A, a series of aptamer variants was prepared with few structural changes (see Supplementary Figure S1). The binding affinity parameters for the variants were determined by a Biacore T200 analysis (see Supplementary Figure S2). The sequence of each aptamer variant is listed in Supplementary Table S1.

Previously, we reported the optimized anti-VEGF_165_ and anti-IFNγ aptamers containing two unnatural Ds bases (V-Apt1 (DD) and I-Apt1 (DD) in Figure 1), in which an extraordinarily stable mini-hairpin sequence, CGCG(A/T)AGCG, was introduced (22,25,26). To prepare each set of aptamers with various *K*_D_ values, we chemically synthesized their aptamer variants by replacing one or two Ds bases with A bases (Figure 2 and Supplementary Table S1). To use these aptamers as capture or detector agents in ELISA/ELONA assays, we conjugated biotin to position 5 of the T (U) base in the tri-nucleotide loop of the mini-hairpin DNA sequence (27,28) at the 3′-end, where the modification would not disturb the aptamer's binding to the target (25,26). To increase the stabilities against thermal denaturation and enzymatic degradation, the mini-hairpin DNA sequence (27,28) was also introduced to the internal hairpin regions of the I-Apt aptamers (25).

The secondary structures of the aptamers shown in Figure 1 were predicted by the co-variation data from the doped ExSELEX and by chemical and enzymatic probing (22). Our V-Apt1 data imply that each site of positions 11–26 and positions 32–45 binds to each molecule of the VEGF_165_ dimer, and thus the V-Apt series molecules are bivalent aptamers, like antibodies. The I-Apt series bind to IFNγ as a 1-to-1 complex as univalent binders, and our data indicate that the four G-tracts in the loop region of I-Apt form a G-quartet structure (data not shown).

First, we measured the binding parameters (*k*_on_, *k*_off_, and *K*_D_) of each aptamer variant to the target proteins by an SPR analysis (Supplementary Figure S1). Figure 2 illustrates the distribution of the binding affinities (*K*_D_ values) for the tested aptamer variants. In the anti-VEGF_165_ aptamer variant set, V-Apt2 (AD), in which the unnatural Ds base at position 25 was replaced with A, exhibited a slightly higher *K*_D_ value of 2.0 pM than that of the original V-Apt1 (DD) aptamer (1.6 pM). In contrast, the Ds→A replacement at position 34 in V-Apt3 (DA) significantly increased the *K*_D_ value from 1.6 pM in V-Apt1 (DD) to 58 pM in V-Apt3 (DA). In the anti-IFNγ aptamer variant set, the Ds→A replacement at position 39 greatly affected the affinity of the aptamer variant, as compared to the replacement at position 28. Overall, the Ds→A replacement reduced both of the aptamers’ *k*_on_ and *k*_off_ values.

Next, we measured the thermal stability of these aptamer variants. Previously, we reported that the Ds→A replacement reduced the thermal stability of DNA aptamers, as well as their affinity. For example, the *T*_m_ values of the anti-IFNγ UB-DNA aptamer (without the mini-hairpin sequence) and its Ds→A variant were 37.6 and 33.1°C, respectively (22). This stability difference around 37°C might affect their sensitivity in the ELISA. Therefore, we introduced the mini-hairpin sequence to all of the DNA aptamers and variants. As a result, the *T*_m_ values of I-Apt1 (DD), I-Apt2 (AD), I-Apt3 (DA), and I-Apt4 (AA) increased to 66, 63.5, 64 and 60°C, respectively. The *T*_m_ values of the V-Apt series also increased to 81, 79.6, 80, and 78.6°C for V-Apt1 (DD), V-Apt2 (AD), V-Apt3 (DA) and V-Apt4 (AA), respectively. All of the melting curves below 40°C were quite similar, indicating that all of the aptamer variants form similar structures under the ELISA/ELONA or other aptamer-usage conditions (Supplementary Figure S2).

Finally, we prepared two sets of thermally stable aptamer variants with various *K*_D_ values, ranging from 1.6 pM to 0.16 nM targeting VEGF_165_ as a bivalent binder and from 13 pM to 16 nM targeting IFNγ as a univalent binder (Figure 2). The natural-base-DNA aptamers, V-Apt4 (AA) and I-Apt4 (AA), with *K*_D_ values of 0.16 and 16 nM, respectively, are representatives of the conventional DNA aptamers. These sets can be used as molecular affinity rulers.

### Gel-mobility shift assay using the molecular affinity rulers

**Figure 3.**
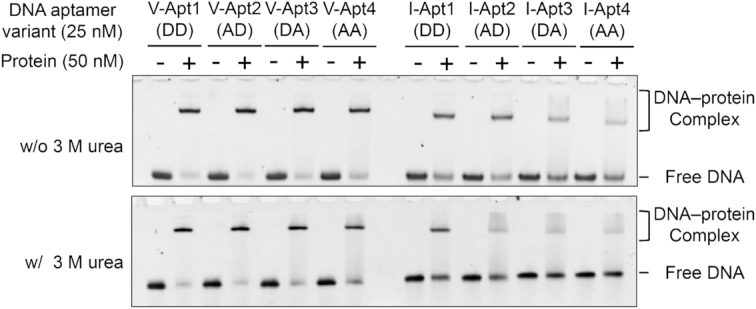
Gel-mobility shift assays of the aptamer−protein complexes using anti-VEGF_165_ and anti-IFNγ aptamer variants. The complexes between each aptamer variant and its target protein were separated from the free aptamer on 8% polyacrylamide gels in the absence and presence of 3 M urea (the upper and lower panels, respectively). The DNA bands on the gels were stained with SYBR Gold and detected by a bioimager.

We demonstrated the properties of the molecular affinity rulers by estimating the detection sensitivity of aptamer−protein complexes by a conventional gel-mobility shift assay. Each target protein was mixed with each aptamer or the variant, and the mixture was electrophoresed under gel conditions in the absence or presence of 3 M urea (Figure 3). In general, the V-Apt series yielded larger shifted bands, as compared to those of the I-Apt series. In both sets, the aptamers with *K*_D_ values less than hundreds of pM exhibited clear shifted bands. Under the 3 M urea conditions, the shifted band densities of not only I-Apt3 (DA) and I-Apt4 (AA) but also I-Apt2 (AD) were significantly reduced. The 3 M urea conditions destabilize the aptamer structures as well as the interactions with their target proteins. Since the *T*_m_ values of the I-Apt series were lower than those of the V-Apt series, the 3 M urea treatment might affect I-Apt more than V-Apt. Overall, the densities of the shifted bands correlated with the aptamer affinities and thermal stabilities, and DNA aptamers with *K*_D_ values less than 10^−9^ M gave clear shifted bands under the native gel conditions.

### Detection of target proteins using aptamer variants as primary detector agents in ELONA

**Figure 4.**
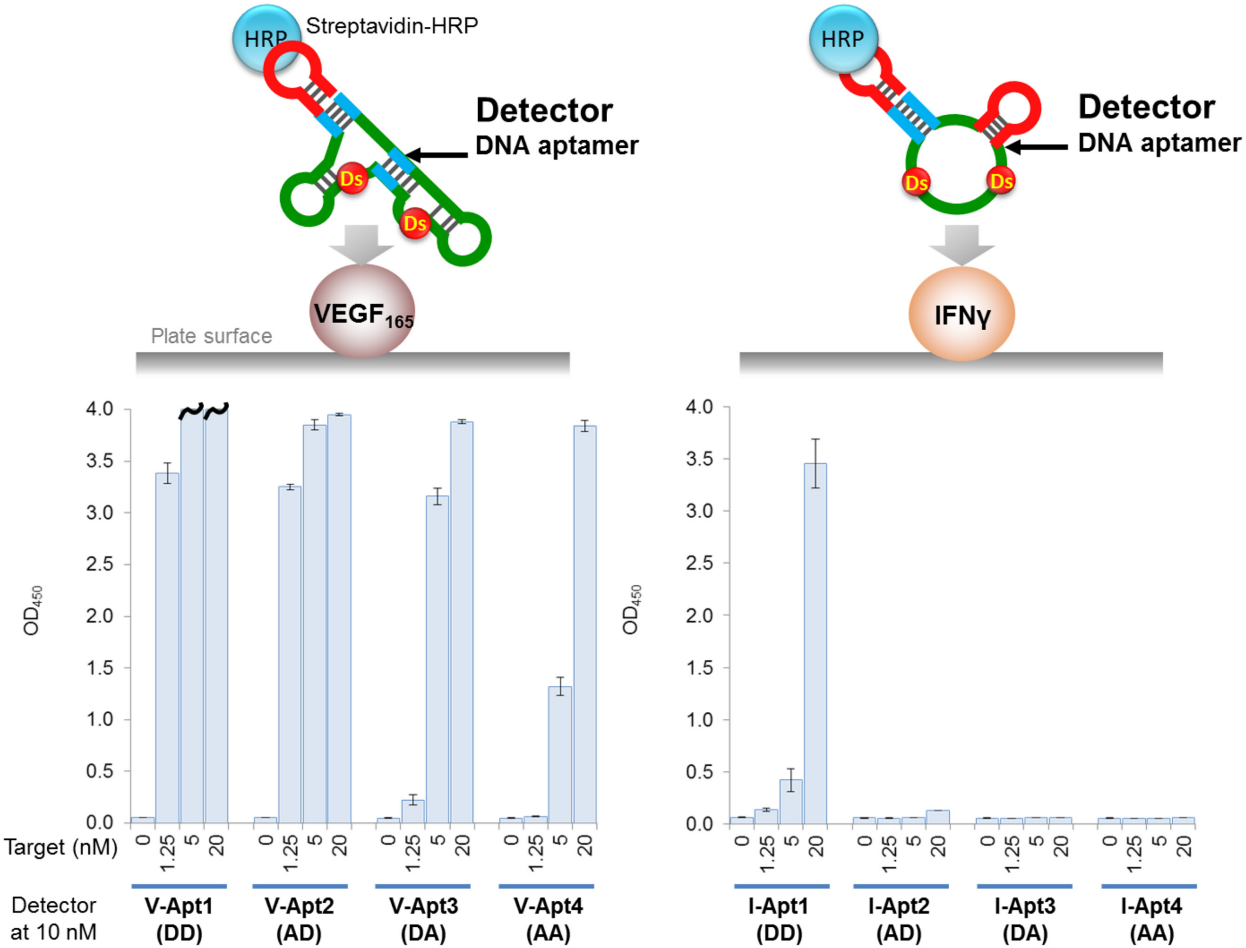
Detection of the target proteins using aptamer variants as the primary detector agents in ELONA. The target was directly immobilized on the plate surface by an incubation with the target solution (100 μl per well) at each indicated concentration, in the presence of 1 μg/ml of BSA in 0.1 M carbonate buffer (pH 9.6), for 2 h. After the incubation, the plate surfaces were further coated with blocking solution (1% BSA in 1× d-PBS(–), 300 μl per well) for 2 h. After the blocking reaction, 100 μl of the detector solution (10 nM each aptamer derivative in 1× binding buffer) was added to each well and then the binding reaction was performed for 30 min. After the incubation, 100 μl of the secondary detector solution (50 ng/ml HRP-conjugated streptavidin in 1× binding buffer) was added to each well, and then incubated for 30 min. After washing the wells, the TMB reaction (100 μl per well) was performed for 5 min (left, VEGF_165_) or 15 min (right, IFNγ). The sample size is two per each combination set, and the error bars represent one standard deviation. The bars with wavy lines indicate that at least one of the two sample wells showed overflow (OD_450_ > 4.000).

Prior to performing the sandwich method, we examined one of the ELONA formats, in which the target proteins are directly immobilized on the plate surface, each biotinylated aptamer variant is added to bind to the targets, and the targets are detected using the streptavidin–HRP conjugate reagent for the colorimetric output of TMB (Figure 4). All of the anti-VEGF_165_ aptamer variants detected the target protein at different protein concentrations, but with varying detection sensitivities (Figure 4, left graph). For example, at a target concentration of 5 nM, V-Apt1 (DD) showed the highest binding signal, followed by V-Apt2 (AD), V-Apt3 (DA) and then V-Apt4 (AA). The order correlated well with the binding capability of each aptamer variant (Figure 2).

In the IFNγ detection with the same method, only I-Apt1 (DD) detected the immobilized IFNγ with reasonable signal sensitivity, while the other Ds→A variants hardly detected the target (Figure 4, right graph). Only a 20 nM target concentration of I-Apt2 (AD) marginally detected IFNγ. Overall, the sensitivities of the aptamer variant series correlated with their binding affinities (*K*_D_ values): high-affinity bivalent aptamers (V-Apt) with *K*_D_ values less than nM to target dimeric proteins, and univalent aptamers (I-Apt) with *K*_D_ values less than several 10 pM to target monomeric proteins, were required for practical use. The tendencies were very similar to those obtained using antibodies: antibody 26503 (*K*_D_ = 58 pM) efficiently detected VEGF_165_, and IFNγ was detectable by antibody B133.5 (*K*_D_ = 66 pM), but less by antibody 2G1 (*K*_D_ = 0.28 nM) (Supplementary Figures S1 and S3). In general, the sensitivity of this method via the direct immobilization of target molecules is low and its application is limited, and thus, we next examined the sandwich-type method using aptamer–antibody combinations.

### Monoclonal antibodies and aptamers as a pair for sandwich-type target detection

We tested sandwich-type ELISA/ELONA formats using a monoclonal antibody (mAb) and our aptamer variant as a pair. To find suitable pairs of antibodies and our aptamer variants, we screened commercially available monoclonal antibodies (mAbs): 26503, VG76e, 16F1 and VG1 for VEGF_165_ and B133.5 and 2G1 for IFNγ, by a gel-mobility shift assay in combination with V-Apt1 (DD) and I-Apt1 (DD) (Supplementary Figure S4). In the gel-mobility shift assay, we detected the aptamers by staining with SYBR Gold, and confirmed that 26503 for VEGF_165_ and 2G1 and B133.5 for IFNγ were present in the higher shifted bands corresponding to the ternary complexes containing the target protein, antibody, and aptamer, relative to the aptamer–protein complex bands shown in Figure 3. Interestingly, 2G1 and B133.5 have previously been used as an antibody–antibody sandwich pair. Thus, the DNA aptamer, I-Apt1 (DD), and two mAbs, 2G1 and B133.5, bind to different areas of IFNγ. For VEGF_165_, only 26503 was obtained in the ternary complex band, and thus it can be used as the pair of V-Apt1 (DD). The use of mAb 26503 in ELISA has been reported in combination with a modified RNA aptamer binding to the heparin-binding domain of VEGF_165_ (35). Since V-Apt1 (DD) also binds to the heparin-binding domain of VEGF_165_, 26503 can be used as the pair of our anti-VEGF_165_ aptamer variants.

For the aptamer evaluation in the sandwich-type ELSA format, we focused on two factors: LOD as detection sensitivity and colorimetric OD_450_ as signal intensity. We determined the LODs by performing each set of independent experiments (refer to Supplementary Figures S7–S10). The signal intensities were evaluated by the OD_450_ values with a series of target protein concentrations (0, 10, 50 and 250 pM) in ELISA.

### Sandwich-type detection of the target proteins using aptamer variants as primary detector agents

**Figure 5.**
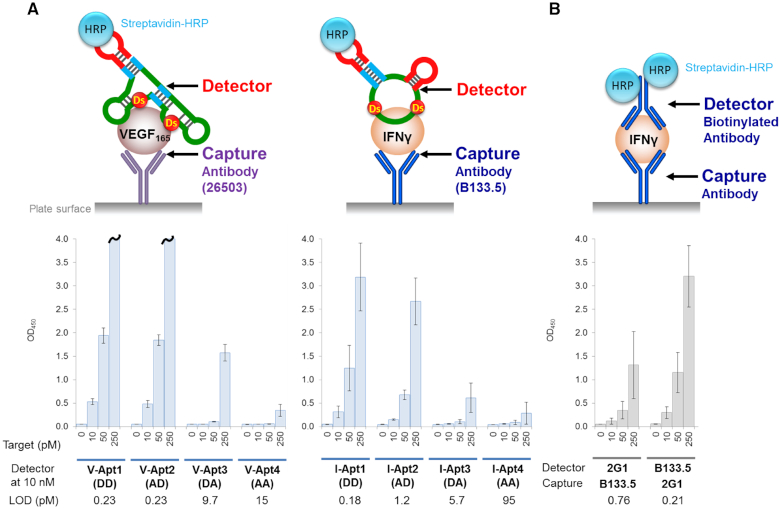
Sandwich-type detection by each aptamer variant as the detector agent in the combination of the pairing monoclonal antibody (mAb) as the capture agent (**A**) and by the antibody–antibody pair (**B**). The target was indirectly immobilized on the plate surface via each corresponding mAb as the capture agent, by an incubation with the target solution (100 μl per well, in 1 × binding buffer) at each indicated concentration. After the indirect immobilization, 100 μl of the detector solution (10 nM each aptamer variant or biotinylated mAb in 1× binding buffer) was added to each well and then the binding reaction was performed for 30 min. After the incubation, 100 μl of the secondary detector solution (50 ng/ml HRP-conjugated streptavidin in 1× binding buffer) was added to each well, followed by an incubation for 30 min. After washing the wells, the TMB reaction (100 μl per well) was performed for 15 min. The sample size is two per each combination set, and the error bars represent one standard deviation. The bars with wavy lines indicate that at least one of the two sample wells showed overflow (OD_450_ > 4.000).

In the sandwich-type ELISA, we first used the mAbs (26503 for VEGF_165_ and B133.5 for IFNγ) as the capture agents for the immobilization of the target proteins and the aptamer variants as the primary detector agents. The target protein solution was added to the immobilized mAbs on the plate, and after washing the complex, each aptamer variant was added, followed by mixing with the Streptavidin–HRP conjugate reagent for the colorimetric detection. The detection sensitivity (LOD) of each aptamer correlated well with its signal intensity (OD_450_). In addition, the detection sensitivities and signal intensity patterns of each target protein (Figure 5) also correlated well with the respective aptamer's binding affinities, similar to the direct ELISA/ELONA assay (Figure 4), but with greatly increased sensitivities. In the VEGF_165_ detection using mAb 26503 as the capture agent, V-Apt1 (DD) and V-Apt2 (AD), which have similar affinities, exhibited nearly the same detection sensitivities (LOD = 0.23 pM). Overall, the aptamer variants (V-Apt4, I-Apt3, and I-Apt4) with *K*_D_ values more than hundreds of pM (>10^−10^ M) exhibited significantly reduced signal intensities. Therefore, when aptamers are used as primary detector agents, the required affinities are lower than 10^−10^ M of *K*_D,_ for the high sensitive ELISA format with LODs lower than several pM and reliable signal intensities. In addition, there was no significant difference between the bivalent V-Apt and univalent I-Apt aptamers in terms of their detection sensitives and signal intensities.

The sensitivities (LOD = 0.21–0.76 pM) of antibody–antibody pairs using 2G1 and B133.5 to IFNγ (Figure 5B) were as high as those (LOD = 0.18–0.23 pM) of aptamer–antibody pairs using V-Apt1, V-Apt2 or I-Apt1 (Figure 5A), indicating that the higher affinities (*K*_D_ of lower than ten pM) of the aptamers were required for the sufficient sensitivities, as compared to those of the antibodies (*K*_D_ = 66–280 pM). In addition, the signal intensity (OD_450_) for the VEGF_165_ detection, using V-Apt3 (58 pM) as the detector agent and mAb 26503 (58 pM) as the capture agent, was similar to that for the IFNγ detection using the antibody−antibody combination, in which the mAbs 2G1 (280 pM) and B133.5 (66 pM) were used as the detector and capture agents, respectively. Similarly, the intensity for the VEGF_165_ detection using V-Apt1 or 2 (1.6 or 2.0 pM) as the detector agents and mAb 26503 (58 pM) as the capture agent was similar to that for the IFNγ detection using mAb B133.5 (66 pM) as the detector agent and mAb 2G1 (280 pM) as the capture agent. These results indicate that around 5−30-fold higher affinities (58 versus 280 pM and 2 versus 66 pM) of the DNA aptamers are required for their use as the detector agents, as compared to the antibodies, when focusing on the affinities of the detector agents.

In ELISA/ELONA using the combination of the aptamer as the detector agent and the antibody as the capture agent, the antibody affinities were also important for the sensitivities. B133.5 (66 pM) as a capture agent exhibited higher sensitivity for IFNγ detection, as compared to that of 2G1 (280 pM), in which the synergetic effect with the aptamer affinities was observed (Supplementary Figure S5). In addition, increasing the concentrations of the low-affinity detector aptamers slightly improved the signal intensities (Supplementary Figures S6 and S7).

### Sandwich-type detection of the target proteins using aptamer variants as capture agents

**Figure 6.**
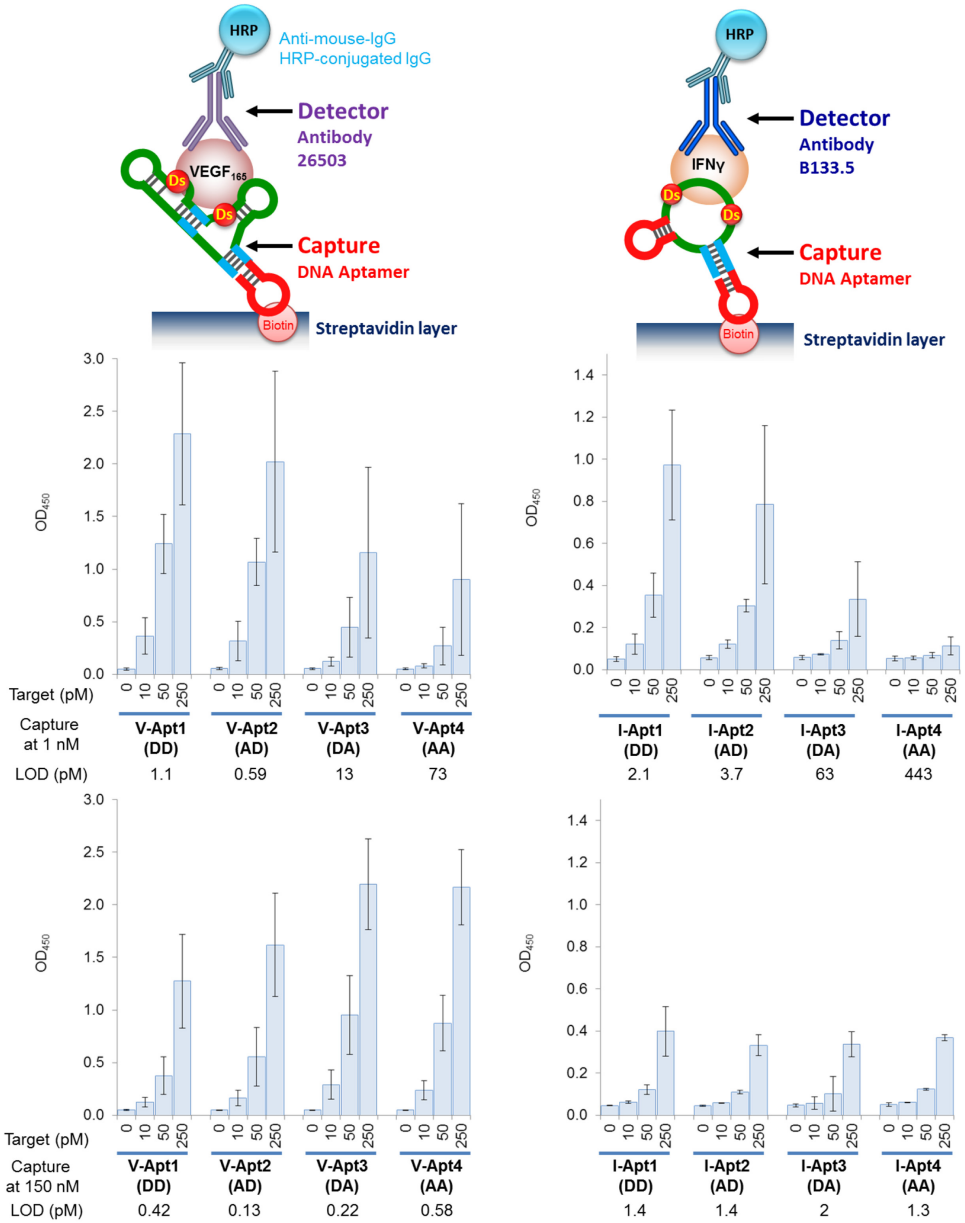
Sandwich-type detection by each aptamer variant as a capture agent. The aptamer was immobilized on the plate surface coated with streptavidin, by an incubation for 30 min at 1 nM or 150 nM in 1× binding buffer (100 μl per well). The target binding was then performed by an incubation with the target solution (100 μl per well, in 1× binding buffer) at each indicated concentration. After the aptamer-target binding, 100 μl of the detector solution (10 nM each monoclonal antibody in 1× binding buffer) was added to each well and the plate was incubated for 30 min. After the incubation, 100 μl of the secondary detector solution (50 ng/ml HRP-conjugated anti-mouse IgG antibody in 1× binding buffer) was added to each well, followed by an incubation for 30 min. After washing the wells, the TMB reaction (100 μl per well) was performed for 15 min. The sample size is two per each combination set, and the error bars represent one standard deviation.

Next, we examined the effectiveness of the combination of the aptamer variants as the capture agents and the antibodies as the detector agents. Each aptamer solution (1 or 150 nM) was dispensed onto a plate coated with a streptavidin layer for immobilization. After washing the plate to remove the unbound aptamers, the target protein was added. After another wash of the plate, the primary detector mAb was added, and the signal was detected using the secondary anti-mouse IgG HRP-conjugated antibody (Figure 6).

When low concentrations (1 nM) of the aptamer solution were used for immobilization, both VEGF_165_ and IFNγ could be reliably detected by the respective aptamer variants with *K*_D_ values lower than several tens of pM (Upper panels in Figure 6). The detection sensitivities and signal intensities of each aptamer variant again correlated well with the respective aptamer affinities. Surprisingly, when higher concentrations (150 nM) of the aptamer solution were used for immobilization, the opposite tendency in signal intensities was observed in the affinity-detection relationship (Lower panels in Figure 6). Most of the DNA aptamers containing Ds bases, including V-Apt1 (DD), V-Apt2 (AD), I-Apt1 (DD) and I-Apt2 (AD), showed reductions in their signal intensities when higher aptamer concentrations were used for immobilization. In contrast, the weakest binders, V-Apt4 (AA) and I-Apt4 (AA), as well as V-Apt3 (DA), showed increased signal intensities when higher concentrations of aptamers were immobilized. Although the LODs of the high-affinity aptamers were identical between the two concentrations (1 and 150 nM), the LODs of the low-affinity aptamers were greatly improved by increasing their concentrations (Supplementary Figures S8 and S10).

**Figure 7.**
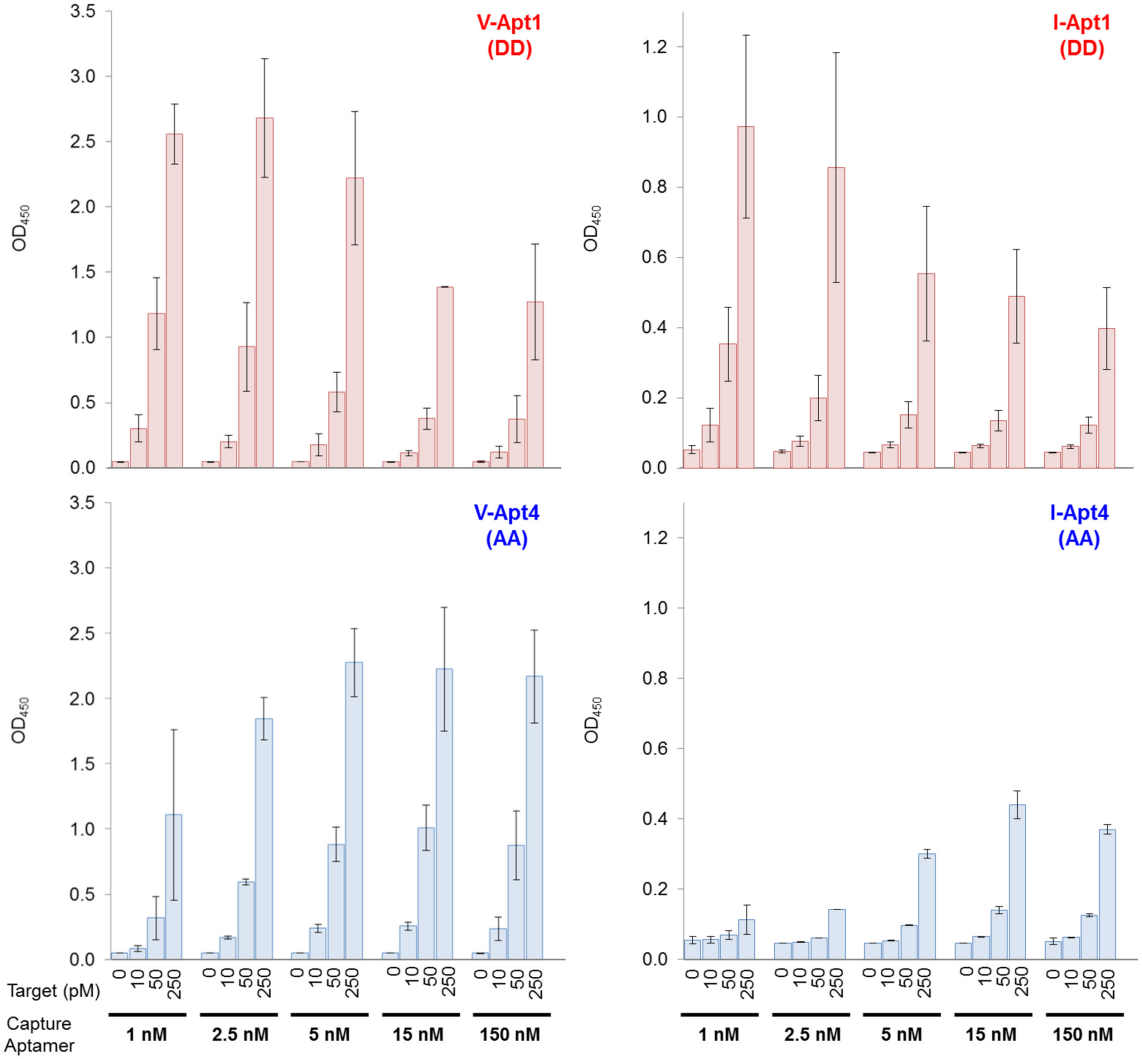
Concentration dependency of each aptamer variant as a capture agent in sandwich-type detection. Different concentrations (0.5–150 nM in 1× binding buffer, 100 μl per well) of the aptamer were immobilized on the plate surface coated with streptavidin, by an incubation for 30 min. The target binding was then performed by an incubation with the target solution (100 μl per well, in 1× binding buffer) at each indicated concentration. After the aptamer–target binding, 100 μl of the detector solution (10 nM each monoclonal antibody, 26503 for VEGF_165_ and B133.5 for IFNγ, in 1× binding buffer) was added to each well and the plate was incubated for 30 min. After the incubation, 100 μl of the secondary detector solution (50 ng/ml HRP-conjugated anti-mouse IgG antibody in 1× binding buffer) was added to each well, followed by an incubation for 30 min. After washing the wells, the TMB reaction (100 μl per well) was performed for 15 min. The sample size is two per each combination set, and the error bars represent one standard deviation.

To investigate this unusual dependency of the immobilized aptamer sensitivities and intensities on the high concentrations of the immobilized aptamers, we examined the signal intensities of the strongest V-Apt1 (DD) and I-Apt1 (DD) and the weakest V-Apt4 (AA) and I-Apt4 (AA) aptamer variants with various amounts of immobilized aptamers, by preparing the plates using different aptamer concentrations ranging from 1 nM to 150 nM (Figure 7). The low-affinity aptamers, V-Apt4 (AA) and I-Apt4 (AA), showed the usual tendency: their signal intensities increased with higher immobilized aptamer densities, although the optimized concentrations of the aptamers for the plate preparation were ∼5 nM for V-Apt4 (AA) and ∼15 nM for I-Apt4 (AA) (Lower panels in Figure 7). In contrast, the signal intensities of the high-affinity Ds-DNA aptamers, V-Apt1 (DD) and I-Apt1 (DD), decreased with increasing immobilized aptamer densities (Upper panels in Figure 7). The optimized concentrations of these aptamers for the plate preparation were ∼1 nM for V-Apt1 (DD) and I-Apt1 (DD).

We presumed that this unusual phenomenon of the high-affinity Ds-DNA aptamers resulted from the intermolecular interactions between the immobilized aptamers, which prevented their binding to the target proteins. Although the aptamer-aptamer interactions were observed with high concentrations of the conventional aptamers, such as V-Apt4 (AA) and I-Apt4 (AA), the interactions between the hydrophobic Ds bases in each Ds-DNA aptamer were enhanced in an aqueous solution when the aptamer-aptamer distances were shorter on the two-dimensional plate. In addition, streptavidin has four binding sites for biotin, and thus the biotinylated aptamers might be immobilized quite closely to each another.

**Figure 8.**
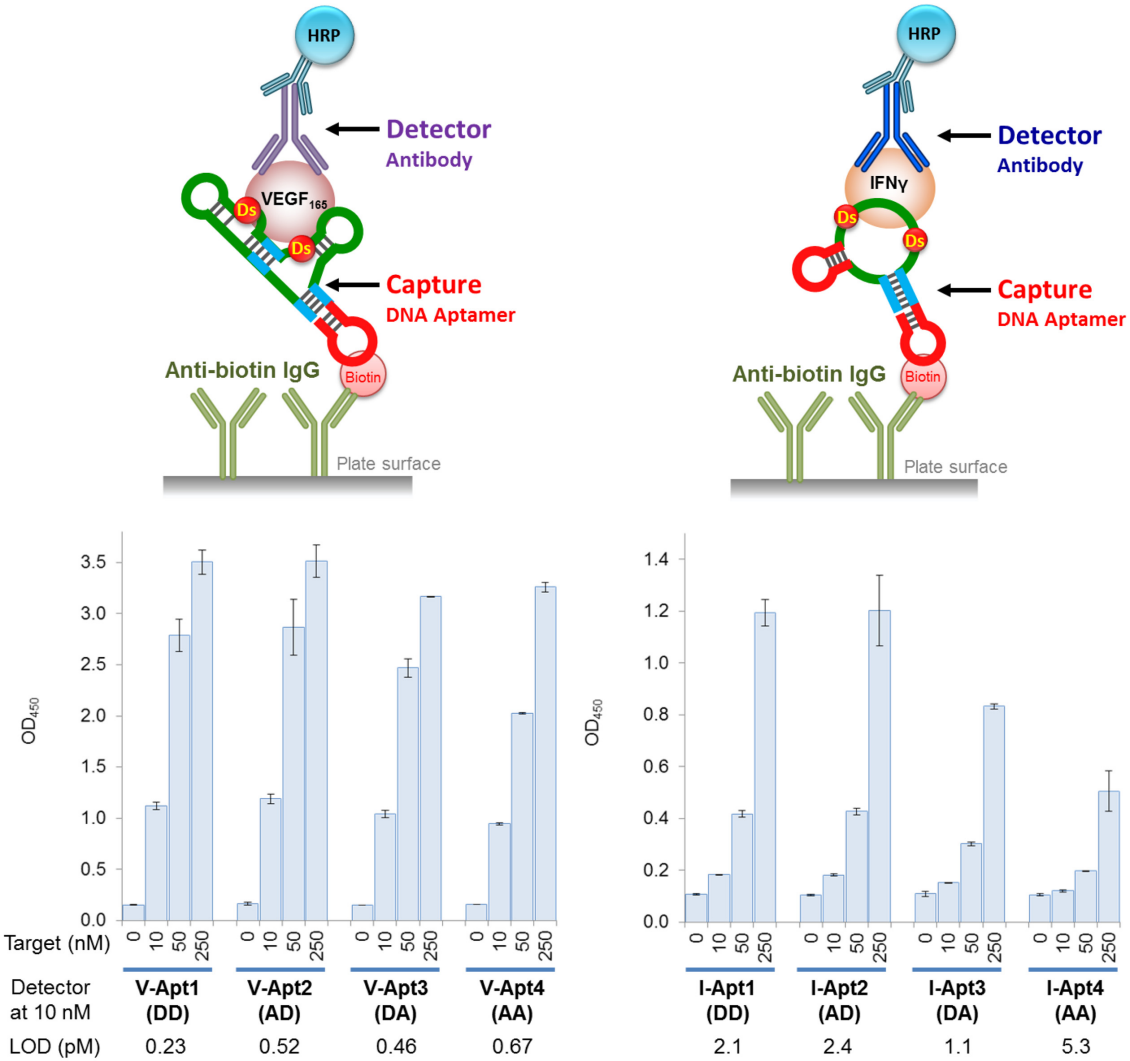
Sandwich-type detection by each aptamer variant as a capture agent, immobilized via anti-biotin IgG. The aptamer (150 nM in 1× binding buffer) was immobilized on the plate surface coated with anti-biotin IgG, by an incubation for 30 min (100 μl per well). The target binding was then performed by an incubation with the target solution (100 μl, in 1× binding buffer per well) at each indicated concentration. After the aptamer-target binding, 100 μl of the detector solution (10 nM each monoclonal antibody in 1× binding buffer) was added to each well and the plate was incubated for 30 min. After the incubation, 100 μl of the secondary detector solution (50 ng/ml HRP-conjugated anti-mouse IgG antibody in 1× binding buffer) was added to each well, followed by an incubation for 30 min. After washing the wells, the TMB reaction (100 μl per well) was performed for 15 min. The sample size is *n* = 2, and the error bars represent one standard deviation.

Based on this presumption, we improved the immobilization method by coating the plates with anti-biotin IgG, instead of streptavidin. Although IgG has two binding sites, the size of the IgG (∼150 kDa) is larger than that of the tetrameric streptavidin (53 kDa). Thus, the biotinylated aptamers that bind to the anti-biotin IgG have sufficient distance from one another, which might prevent the intermolecular aptamer-aptamer interaction. We immobilized the aptamers using 150 nM aptamer solutions on the anti-biotin-IgG-coated plates. As expected, this immobilization method greatly increased the signal intensities of all of the aptamers (Figure 8 and Supplementary Figures S9 and S10). The results also revealed that the capture aptamers in this immobilization method were more tolerant of wider affinity ranges, as compared the detector aptamers in the ELISA/ELONA system. This method greatly improved the LODs even for the low-affinity aptamers, such as V-Apt4 (AA), I-Apt3 (DA) and I-Apt4 (AA), with *K*_D_ values ranging 0.16−16 nM. In addition, the ELISA format using the bivalent V-Apt aptamers exhibited higher detection sensitivities and signal intensities than that using the univalent I-Apt aptamers, when these aptamers were used as capture agents.

We also examined the concentration dependency of the monoclonal antibodies, when used as the capture agents. For the experiments, we tested the combinations of the capture antibodies and the detector aptamers, 26503 and V-Apt1 (DD) for VEGF_165_ and B133.5 and I-Apt1 (DD) for IFNγ. As shown in Supplementary Figure S11, the tendency was similar to that of the conventional aptamers, V-Apt4 (AA) and I-Apt4 (AA). In both assays, the signal increased with higher concentrations of the capture antibody, from 2.5 to 20 nM, showing that the signal intensities increase along with higher concentrations of the capture agent.

Finally, we examined the practical utility of the ELISA/ELONA system using our affinity molecular rulers for blood tests. In diagnostics to detect some antigens in human fluids, human serum or plasma are commonly used as test samples. If the samples include some binders to the antigen (specific or non-specific, including antibodies to the antigens), then the detection of the antigens might be competitively inhibited (43). Since all of our preliminary experiments were performed in buffer, we examined the detection of the V-Apt and I-Apt sets as the capture agents in the presence of human serum (10%). In the experiments, each biotinylated aptamer was immobilized on the streptavidin-coated plate using the low aptamer concentration solution (1 nM). The high-affinity Ds-DNA aptamers, V-Apt1 (DD), V-Apt2 (AD) and I-Apt1 (DD), were robust under the serum conditions (Supplementary Figure S12). However, using the low-affinity aptamers, the 10% human serum conditions reduced the signal intensities in ELISA.

## DISCUSSION

We developed molecular affinity rulers: two sets of Ds-DNA aptamer variants with a diverse range of affinities to the same area on the target proteins. These molecular affinity rulers can easily be prepared by replacing UBs with natural bases in optimized UB-DNA aptamers. Using the molecular affinity rulers, we examined the relationship between the aptamers’ affinities and their detection sensitivities in ELISA/ELONA formats. The detection sensitivities correlated well with the affinities (*K*_D_ values) of the aptamer variants. As the detector agents in ELISA/ELONA, aptamers with *K*_D_ values lower than several tens of pM are necessary for highly sensitive detection, but as the capture agents, aptamers with several nM *K*_D_ values could be used when the immobilization density was optimized. For the evaluation, we should consider both of these two factors, detection sensitivities (LOD) and signal intensities (OD_450_). When using I-Apt3 as detector agents, the ELISA format exhibited a relatively high LOD (5.7 pM), but the signal intensities were not reliable in view of the errors (Figure 5).

A similar tendency in the relationship between the aptamer affinities and the ELISA sensitivities/intensities was also found in the antibody-antibody pairs using 2G1 (280 pM) and B133.5 (66 pM) for IFNγ detection. The detection sensitivity when using 2G1 as the capture agent and B133.5 as the detector agent was higher than that using B133.5 as the capture agent and 2G1 as the detector agent (Figure 5 and Supplementary Figures S7 and S10). Thus, in the ELISA/ELONA format, stronger and weaker binders should be chosen as the detector and capture agents, respectively.

The molecular affinity rulers are also useful to improve the detection systems. As shown in Figures 7 and 8, the improvement of the immobilization method for the DNA aptamers greatly increased the detection sensitivities and signal intensities. When using the aptamers as the capture agents, we found that the immobilization method using the biotinylated aptamers on the anti-biotin-IgG-coated plate prevents the intermolecular aptamer-aptamer interaction, which significantly increases the ELISA/ELONA sensitivities. Using this immobilization method, both high-affinity Ds-DNA aptamers and low-affinity non-UB-DNA aptamers with nM-range *K*_D_ values can be applied to highly sensitive ELISA/ELONA formats as capture agents. In our experiments, we used rabbit anti-biotin IgG for the plate coating, and found that it increased the background levels in comparison with those of the streptavidin-coated plate. Since the increased background was mainly due to the cross binding of the secondary detection antibody (data not shown), the optimization of the secondary detection antibody concentration was required to increase the signal-to-noise ratios (in this case, 1:20 000). Alternatively, the use of a cross-absorbed anti-mouse IgG HRP conjugate would be helpful to reduce the background level further.

When comparing the similar-affinity aptamer variants, such as V-Apt3 (DA) (58 pM) and I-Apt2 (AD) (55 pM), we found that V-Apt3 (DA) exhibited much higher sensitivity than I-Apt2 (AD) for the target detection, especially when directly immobilizing the target proteins or using aptamers as capture agents. The higher sensitivities of V-Apt than those of I-Apt were also observed in the gel-mobility shift assay of the aptamer−protein complexes (Figure 3). The difference results from the different binding modes of the bivalent anti-VEGF_165_ and univalent anti-IFNγ aptamers to their target proteins. While one anti-IFNγ aptamer binds to one monomer IFNγ, one anti-VEGF_165_ aptamer might bind to the dimeric structure of VEGF_165_. These results and our improved immobilization method of the DNA aptamers using anti-biotin antibodies support high potential of several types of dimeric aptamer structures, such as aptabodies and oligobodies (44–47). Thus, our molecular affinity rulers are two representatives with different binding mode properties, univalent one-to-one or bivalent one-to-two, between aptamers and target molecules.

In general, the target specificity of nucleic acid aptamers is higher than that of antibodies. However, generating two aptamers that bind to one protein target is not still popular for sandwich-type assay platforms (48–50). Thus, the antibody–aptamer combination could be powerful in specific detection and diagnostic methods. Such methods will require high-affinity DNA aptamers. However, DNA aptamers and their detection devices still have room for further improvement to increase their abilities, such as sensitivity. For this purpose, our evaluation method using the aptamer sets with a diverse range of *K*_D_ values is useful as a molecular affinity ruler for estimating the required aptamer affinities and their device sensitivities for practical uses in various assay formats.

## Supplementary Material

gkz688_Supplemental_Files

## References

[1] Ellington A.D., Szostak J.W. *In vitro* selection of RNA molecules that bind specific ligands. Nature. 1990; 346:818–822. 1697402 10.1038/346818a0

[2] Tuerk C., Gold L. Systematic evolution of ligands by exponential enrichment: RNA ligands to bacteriophage T4 DNA polymerase. Science. 1990; 249:505–510. 2200121 10.1126/science.2200121

[3] McKeague M., McConnell E.M., Cruz-Toledo J., Bernard E.D., Pach A., Mastronardi E., Zhang X., Beking M., Francis T., Giamberardino A. et al. Analysis of in vitro aptamer selection parameters. J. Mol. Evol. 2015; 81:150–161. 26530075 10.1007/s00239-015-9708-6

[4] Wang T., Chen C., Larcher L.M., Barrero R.A., Veedu R.N. Three decades of nucleic acid aptamer technologies: Lessons learned, progress and opportunities on aptamer development. Biotechnol. Adv. 2019; 37:28–50. 30408510 10.1016/j.biotechadv.2018.11.001

[5] Mayer G. The chemical biology of aptamers. Angew. Chem. Int. Ed. Engl. 2009; 48:2672–2689. 19319884 10.1002/anie.200804643

[6] Bruno J.G. Predicting the uncertain future of aptamer-based diagnostics and therapeutics. Molecules. 2015; 20:6866–6887. 25913927 10.3390/molecules20046866 PMC6272696

[7] Shigdar S., Macdonald J., O’Connor M., Wang T., Xiang D., Al Shamaileh H., Qiao L., Wei M., Zhou S.F., Zhu Y. et al. Aptamers as theranostic agents: modifications, serum stability and functionalisation. Sensors (Basel). 2013; 13:13624–13637. 24152925 10.3390/s131013624 PMC3859083

[8] Groher F., Suess B. *In vitro* selection of antibiotic-binding aptamers. Methods. 2016; 106:42–50. 27223401 10.1016/j.ymeth.2016.05.008

[9] Rothlisberger P., Hollenstein M. Aptamer chemistry. Adv. Drug. Deliv. Rev. 2018; 134:3–21. 29626546 10.1016/j.addr.2018.04.007

[10] Hirao I., Kimoto M., Lee K.H. DNA aptamer generation by ExSELEX using genetic alphabet expansion with a mini-hairpin DNA stabilization method. Biochimie. 2018; 145:15–21. 28916151 10.1016/j.biochi.2017.09.007

[11] Sefah K., Yang Z., Bradley K.M., Hoshika S., Jimenez E., Zhang L., Zhu G., Shanker S., Yu F., Turek D. et al. *In vitro* selection with artificial expanded genetic information systems. Proc. Natl. Acad. Sci. U.S.A. 2014; 111:1449–1454. 24379378 10.1073/pnas.1311778111 PMC3910645

[12] Thirunavukarasu D., Chen T., Liu Z., Hongdilokkul N., Romesberg F.E. Selection of 2′-Fluoro-Modified aptamers with optimized properties. J. Am. Chem. Soc. 2017; 139:2892–2895. 28218835 10.1021/jacs.6b13132

[13] Gold L., Ayers D., Bertino J., Bock C., Bock A., Brody E.N., Carter J., Dalby A.B., Eaton B.E., Fitzwater T. et al. Aptamer-based multiplexed proteomic technology for biomarker discovery. PLoS One. 2010; 5:e15004. 21165148 10.1371/journal.pone.0015004 PMC3000457

[14] Gold L., Walker J.J., Wilcox S.K., Williams S. Advances in human proteomics at high scale with the SOMAscan proteomics platform. N. Biotechnol. 2012; 29:543–549. 22155539 10.1016/j.nbt.2011.11.016

[15] Sun B.B., Maranville J.C., Peters J.E., Stacey D., Staley J.R., Blackshaw J., Burgess S., Jiang T., Paige E., Surendran P. et al. Genomic atlas of the human plasma proteome. Nature. 2018; 558:73–79. 29875488 10.1038/s41586-018-0175-2 PMC6697541

[16] Ruckman J., Green L.S., Beeson J., Waugh S., Gillette W.L., Henninger D.D., Claesson-Welsh L., Janjic N. 2′-Fluoropyrimidine RNA-based aptamers to the 165-amino acid form of vascular endothelial growth factor (VEGF165). Inhibition of receptor binding and VEGF-induced vascular permeability through interactions requiring the exon 7-encoded domain. J. Biol. Chem. 1998; 273:20556–20567. 9685413 10.1074/jbc.273.32.20556

[17] Ng E.W., Shima D.T., Calias P., Cunningham E.T. Jr, Guyer D.R., Adamis A.P. Pegaptanib, a targeted anti-VEGF aptamer for ocular vascular disease. Nat. Rev. Drug Discov. 2006; 5:123–132. 16518379 10.1038/nrd1955

[18] Drolet D.W., Green L.S., Gold L., Janjic N. Fit for the Eye: Aptamers in ocular disorders. Nucleic Acid Ther. 2016; 26:127–146. 26757406 10.1089/nat.2015.0573 PMC4900223

[19] Pereira R.L., Nascimento I.C., Santos A.P., Ogusuku I.E.Y., Lameu C., Mayer G., Ulrich H. Aptamers: novelty tools for cancer biology. Oncotarget. 2018; 9:26934–26953. 29928493 10.18632/oncotarget.25260 PMC6003562

[20] Nimjee S.M., White R.R., Becker R.C., Sullenger B.A. Aptamers as therapeutics. Annu. Rev. Pharmacol. Toxicol. 2017; 57:61–79. 28061688 10.1146/annurev-pharmtox-010716-104558 PMC6035745

[21] Pan Q., Luo F., Liu M., Zhang X.L. Oligonucleotide aptamers: promising and powerful diagnostic and therapeutic tools for infectious diseases. J. Infect. 2018; 77:83–98. 29746951 10.1016/j.jinf.2018.04.007 PMC7112547

[22] Kimoto M., Yamashige R., Matsunaga K., Yokoyama S., Hirao I. Generation of high-affinity DNA aptamers using an expanded genetic alphabet. Nat. Biotechnol. 2013; 31:453–457. 23563318 10.1038/nbt.2556

[23] Futami K., Kimoto M., Lim Y.W.S., Hirao I. Genetic alphabet expansion provides versatile specificities and activities of Unnatural-Base DNA aptamers targeting cancer cells. Mol. Ther. Nucleic Acids. 2019; 14:158–170. 30594072 10.1016/j.omtn.2018.11.011 PMC6307347

[24] Matsunaga K., Kimoto M., Hirao I. High-affinity DNA aptamer generation targeting von Willebrand factor A1-domain by genetic alphabet expansion for systematic evolution of ligands by exponential enrichment using two types of libraries composed of five different bases. J. Am. Chem. Soc. 2017; 139:324–334. 27966933 10.1021/jacs.6b10767

[25] Matsunaga K., Kimoto M., Hanson C., Sanford M., Young H.A., Hirao I. Architecture of high-affinity unnatural-base DNA aptamers toward pharmaceutical applications. Sci. Rep. 2015; 5:18478. 26690672 10.1038/srep18478 PMC4686876

[26] Kimoto M., Nakamura M., Hirao I. Post-ExSELEX stabilization of an unnatural-base DNA aptamer targeting VEGF165 toward pharmaceutical applications. Nucleic Acids Res. 2016; 44:7487–7494. 27387284 10.1093/nar/gkw619 PMC5009754

[27] Hirao I., Kawai G., Yoshizawa S., Nishimura Y., Ishido Y., Watanabe K., Miura K. Most compact hairpin-turn structure exerted by a short DNA fragment, d(GCGAAGC) in solution: an extraordinarily stable structure resistant to nucleases and heat. Nucleic Acids Res. 1994; 22:576–582. 8127706 10.1093/nar/22.4.576 PMC307846

[28] Yoshizawa S., Kawai G., Watanabe K., Miura K., Hirao I. GNA trinucleotide loop sequences producing extraordinarily stable DNA minihairpins. Biochemistry. 1997; 36:4761–4767. 9125496 10.1021/bi961738p

[29] Engvall E., Perlmann P. Enzyme-linked immunosorbent assay (ELISA). Quantitative assay of immunoglobulin G. Immunochemistry. 1971; 8:871–874. 5135623 10.1016/0019-2791(71)90454-x

[30] Gan S.D., Patel K.R. Enzyme immunoassay and enzyme-linked immunosorbent assay. J. Invest. Dermatol. 2013; 133:e12. 23949770 10.1038/jid.2013.287

[31] Liang M., Klakamp S.L., Funelas C., Lu H., Lam B., Herl C., Umble A., Drake A.W., Pak M., Ageyeva N. et al. Detection of high- and low-affinity antibodies against a human monoclonal antibody using various technology platforms. Assay Drug. Dev. Technol. 2007; 5:655–662. 17939757 10.1089/adt.2007.089

[32] Bobrovnik S.A. Determination of antibody affinity by ELISA. Theory. J. Biochem. Biophys. Methods. 2003; 57:213–236. 14512156 10.1016/s0165-022x(03)00145-3

[33] Hu D., Fry S.R., Huang J.X., Ding X., Qiu L., Pan Y., Chen Y., Jin J., McElnea C., Buechler J. et al. Comparison of surface plasmon resonance, resonant waveguide grating biosensing and enzyme linked immunosorbent assay (ELISA) in the evaluation of a dengue virus immunoassay. Biosensors (Basel). 2013; 3:297–311. 25586260 10.3390/bios3030297 PMC4263579

[34] Green L.S., Jellinek D., Bell C., Beebe L.A., Feistner B.D., Gill S.C., Jucker F.M., Janjic N. Nuclease-resistant nucleic acid ligands to vascular permeability factor/vascular endothelial growth factor. Chem. Biol. 1995; 2:683–695. 9383475 10.1016/1074-5521(95)90032-2

[35] Drolet D.W., Moon-McDermott L., Romig T.S. An enzyme-linked oligonucleotide assay. Nat. Biotechnol. 1996; 14:1021–1025. 9631044 10.1038/nbt0896-1021

[36] Dehghani S., Nosrati R., Yousefi M., Nezami A., Soltani F., Taghdisi S.M., Abnous K., Alibolandi M., Ramezani M. Aptamer-based biosensors and nanosensors for the detection of vascular endothelial growth factor (VEGF): A review. Biosens. Bioelectron. 2018; 110:23–37. 29579646 10.1016/j.bios.2018.03.037

[37] Razmi N., Baradaran B., Hejazi M., Hasanzadeh M., Mosafer J., Mokhtarzadeh A., de la Guardia M. Recent advances on aptamer-based biosensors to detection of platelet-derived growth factor. Biosens. Bioelectron. 2018; 113:58–71. 29729560 10.1016/j.bios.2018.04.048

[38] Akki S.U., Werth C.J. Critical Review: DNA aptasensors, are they ready for monitoring organic pollutants in natural and treated water sources. Environ. Sci. Technol. 2018; 52:8989–9007. 30016080 10.1021/acs.est.8b00558

[39] Seo H.B., Gu M.B. Aptamer-based sandwich-type biosensors. J. Biol. Eng. 2017; 11:11. 28293287 10.1186/s13036-017-0054-7 PMC5346835

[40] Toh S.Y., Citartan M., Gopinath S.C., Tang T.H. Aptamers as a replacement for antibodies in enzyme-linked immunosorbent assay. Biosens. Bioelectron. 2015; 64:392–403. 25278480 10.1016/j.bios.2014.09.026

[41] Wu D., Katilius E., Olivas E., Dumont Milutinovic M., Walt D.R. Incorporation of slow off-rate modified aptamers reagents in single molecule array assays for cytokine detection with ultrahigh sensitivity. Anal. Chem. 2016; 88:8385–8389. 27529794 10.1021/acs.analchem.6b02451

[42] Hirao I., Kimoto M., Mitsui T., Fujiwara T., Kawai R., Sato A., Harada Y., Yokoyama S. An unnatural hydrophobic base pair system: site-specific incorporation of nucleotide analogs into DNA and RNA. Nat. Methods. 2006; 3:729–735. 16929319 10.1038/nmeth915

[43] Fu P., Sun Z., Yu Z., Zhang Y., Shen J., Zhang H., Xu W., Jiang F., Chen H., Wu W. Enzyme linked aptamer assay: based on a competition format for sensitive detection of antibodies to *Mycoplasma bovis* in serum. Anal. Chem. 2014; 86:1701–1709. 24417693 10.1021/ac4042203

[44] Hasegawa H., Taira K.I., Sode K., Ikebukuro K. Improvement of aptamer affinity by dimerization. Sensors (Basel). 2008; 8:1090–1098. 27879754 10.3390/s8021090 PMC3927496

[45] Hianik T., Porfireva A., Grman I., Evtugyn G. Aptabodies - new type of artificial receptors for detection proteins. Protein Pept. Lett. 2008; 15:799–805. 18855752 10.2174/092986608785203656

[46] Heo K., Min S.W., Sung H.J., Kim H.G., Kim H.J., Kim Y.H., Choi B.K., Han S., Chung S., Lee E.S. et al. An aptamer-antibody complex (oligobody) as a novel delivery platform for targeted cancer therapies. J. Control. Release. 2016; 229:1–9. 26956592 10.1016/j.jconrel.2016.03.006

[47] Zhou J., Rossi J. Aptamers as targeted therapeutics: current potential and challenges. Nat. Rev. Drug Discov. 2017; 16:181–202. 27807347 10.1038/nrd.2016.199 PMC5700751

[48] Ochsner U.A., Green L.S., Gold L., Janjic N. Systematic selection of modified aptamer pairs for diagnostic sandwich assays. BioTechniques. 2014; 56:125–133. 24641476 10.2144/000114134

[49] Ahmad Raston N.H., Nguyen V.T., Gu M.B. A new lateral flow strip assay (LFSA) using a pair of aptamers for the detection of Vaspin. Biosens. Bioelectron. 2017; 93:21–25. 27916536 10.1016/j.bios.2016.11.061

[50] Fang L.X., Huang K.J., Liu Y. Novel electrochemical dual-aptamer-based sandwich biosensor using molybdenum disulfide/carbon aerogel composites and Au nanoparticles for signal amplification. Biosens. Bioelectron. 2015; 71:171–178. 25909336 10.1016/j.bios.2015.04.031

