# Supplementary material for: Molecular affinity rulers: systematic evaluation of DNA aptamers for their applicabilities in ELISA
Source: Nucleic Acids Res. 2019 Aug 8;47(16):8362–74. doi: 10.1093/nar/gkz688 (PMC6895277; doi:10.1093/nar/gkz688)
Supplement: gkz688_Supplemental_Files [file gkz688_supplemental_files.zip › 20190709 revised SI Kimoto et al final ver.pdf]

## SUPPLEMENTARY INFORMATION

# Molecular affinity rulers: systematic evaluation of DNA aptamers for their applicabilities

Michiko Kimoto[1], Yun Wei Shermane Lim[1,2] and Ichiro Hirao[1,]*

[1] Institute of Bioengineering and Nanotechnology, 31 Biopolis Way, The Nanos, #07-01, Singapore 138669, Singapore
[2] NUS High School of Mathematics and Science, 20 Clementi Avenue 1, Singapore 129957, Singapore

**Supplementary Figure S1.** Binding affinities of DNA aptamer variants and monoclonal antibodies to each target by a Biacore T200 analysis at 25°C.

**Supplementary Figure S2.** Thermal stabilities of DNA aptamer variants.

**Supplementary Figure S3.** Detection of the target proteins using monoclonal antibodies as the primary detector agents.

**Supplementary Figure S4.** Gel shift assay to find sandwich aptamer-antibody pairs for target detection.

**Supplementary Figure S5.** Sandwich-type detection by each anti-IFNγ aptamer variant as the primary detector agent in the combination of the pairing monoclonal antibody (2G1 or B133.5) as the capture agent.

**Supplementary Figure S6.** Sandwich-type detection by each aptamer variant as a detector agent in the combination of the pairing monoclonal antibody (mAb) as the capture agent.

**Supplementary Figure S7.** Determination of limit of detection (LOD) targeting $VEGF_{165}$ and IFNγ by a sandwich-type ELISA, using antibodies as capture agents.

**Supplementary Figure S8.** Determination of limit of detection (LOD) targeting $VEGF_{165}$ and IFNγ by a sandwich-type ELISA, using aptamers as capture agents on the plates coated with streptavidin.

**Supplementary Figure S9.** Determination of limit of detection (LOD) targeting $VEGF_{165}$ and IFNγ by a sandwich-type ELISA, using aptamers as capture agents on the plates coated with anti-biotin IgG.

**Supplementary Figure S10.** Summary of the limit of detections (LODs) targeting $VEGF_{165}$ and IFNγ by a sandwich-type ELISA using Ds-DNA aptamers and their variants as detector or capture agents in combination with each cognate antibody.

**Supplementary Figure S11.** Concentration dependency of monoclonal antibodies as capture agents in the sandwich-type detection.

**Supplementary Figure S12.** Sandwich-type detection by each aptamer variant as a capture agent in the presence of human serum (HS).

**Supplementary Table S1.** DNA sequences used in this study.

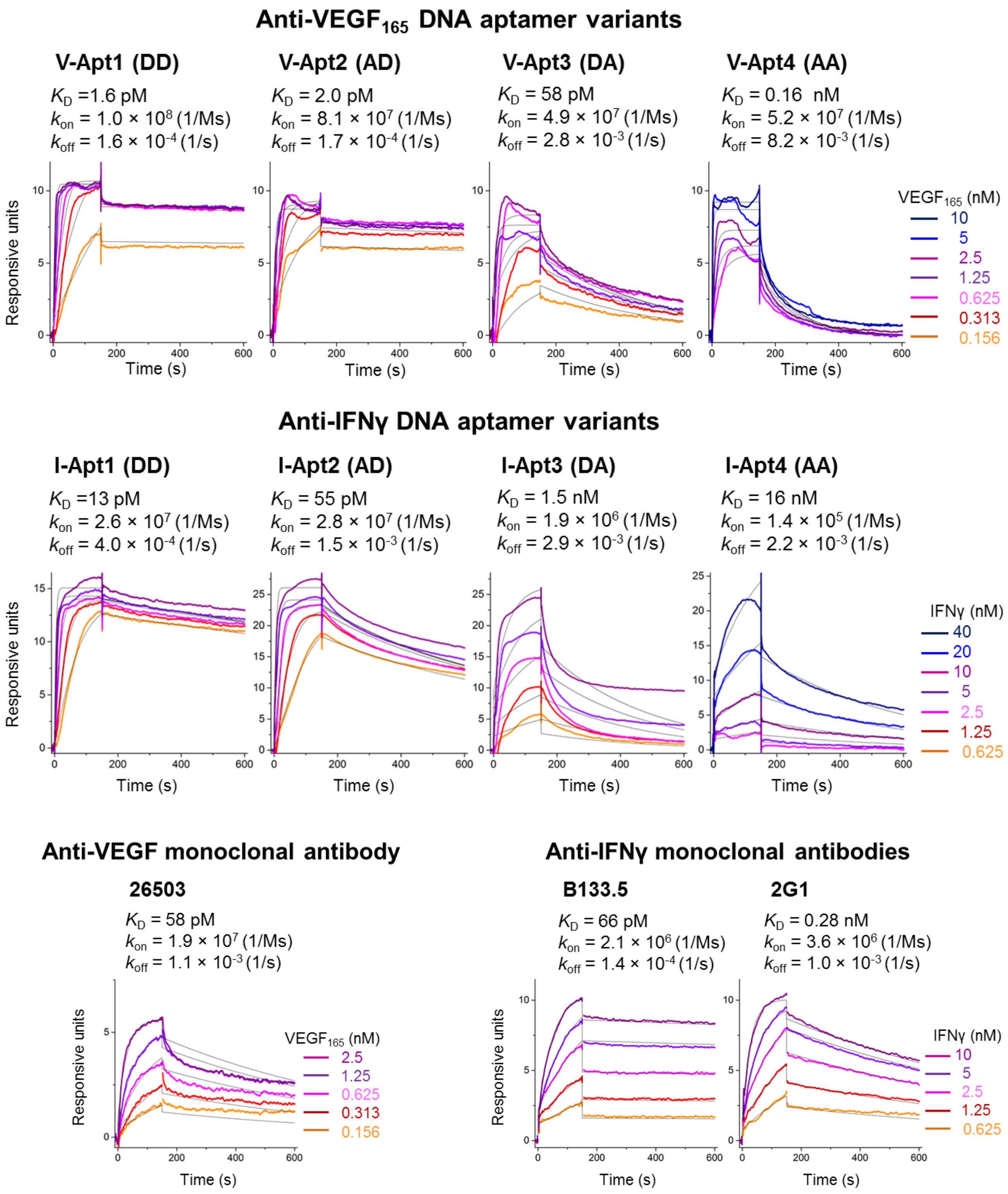


**Supplementary Figure S1. Binding affinities of DNA aptamer variants and monoclonal antibodies to each target by a Biacore T200 analysis at 25°C.** Running buffer: (for $VEGF_{165}$ binding) 1× PBS (1 mM $KH_2PO_4$, 3 mM $Na_2HPO_4$, and 155 mM NaCl, pH 7.4) supplemented with 0.05% Nonidet-P 40; (for IFNγ binding) 1× PBS (1 mM $KH_2PO_4$, 3 mM $Na_2HPO_4$, and 205 mM NaCl, pH 7.4) supplemented with 0.05% Nonidet P-40. Flow rate: 100 µl/min. Injection (association) time: 150 sec. Dissociation time: 450 sec. The $K_D$ values were determined through 1:1 global fitting with the Biacore T200 evaluation software, by using more than two or three injection data at each target protein concentration. To avoid the mass-transfer effect as much as possible, the amounts of aptamers and monoclonal antibodies were kept at low levels. Representative association and dissociation curves with fitting (gray lines) are shown. Regeneration was performed with a 5-sec injection of 50 mM NaOH (for aptamers) or 10 mM Glycine-HCl pH 2.5 (for antibodies) , followed by a 10-min equilibration with each running buffer.

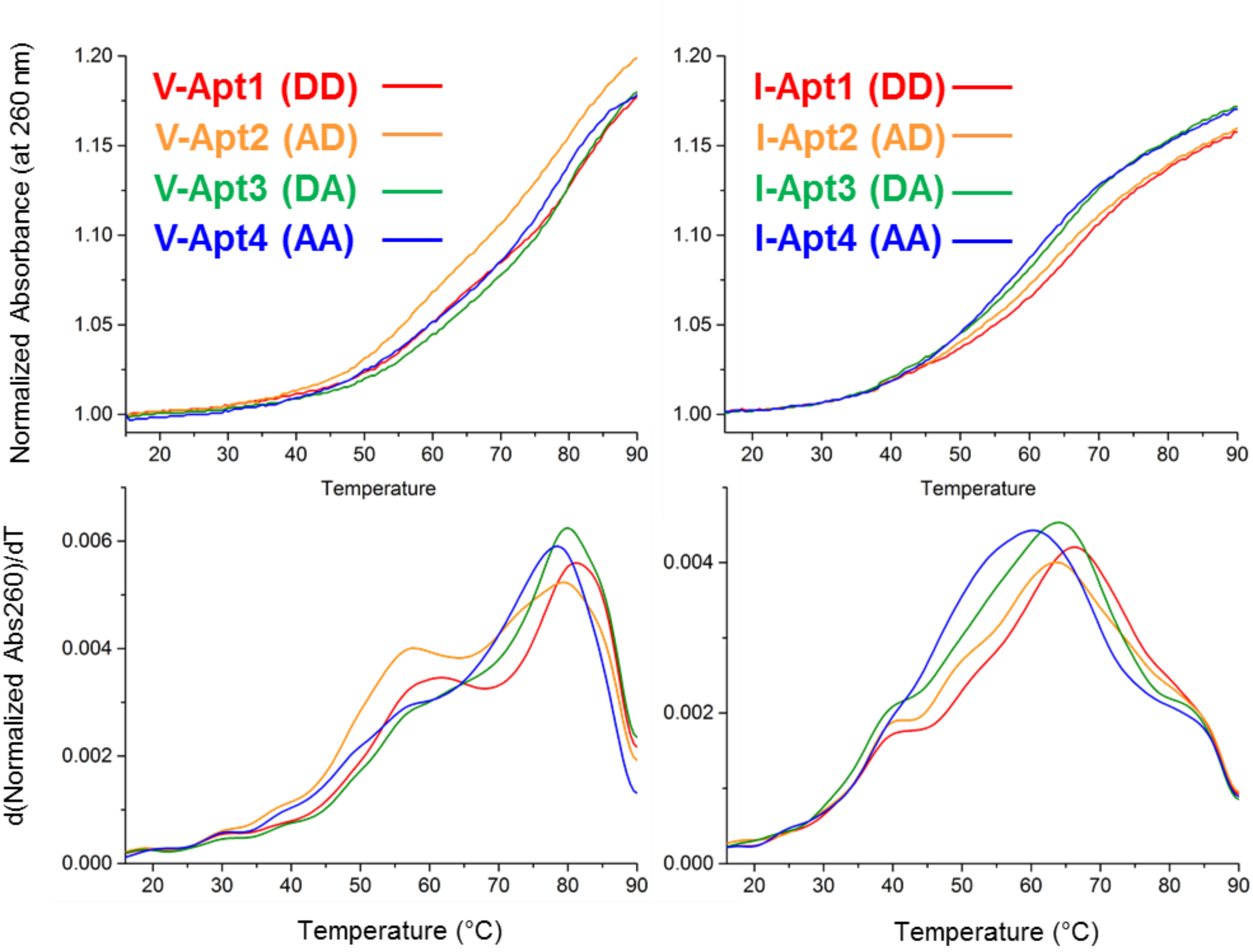


**Supplementary Figure S2. Thermal stabilities of DNA aptamer variants.** UV absorbance melting profiles of anti-$VEGF_{165}$ and anti-IFNγ aptamer variants were monitored with a SHIMADZU UV-2600 spectrometer equipped with a temperature controller. The absorbances of each sample (2 μM in 1× PBS (1 mM $KH_2PO_4$, 3 mM $Na_2HPO_4$, and 155 mM NaCl, pH 7.4)) were measured at 260 nm from 15 to 90°C, at a heating rate of 0.5°C/min. The normalized absorbances at 15°C were plotted against the temperature. The melting temperatures were calculated by the first derivative of each melting curve, using the IGOR Pro software (WaveMetrics, Inc.) The first derivatives of the absorbance are plotted against the temperature in the lower panels. The melting profiles indicate that the replacement of each Ds base with the natural adenine base did not cause large structural changes at physiological temperatures.

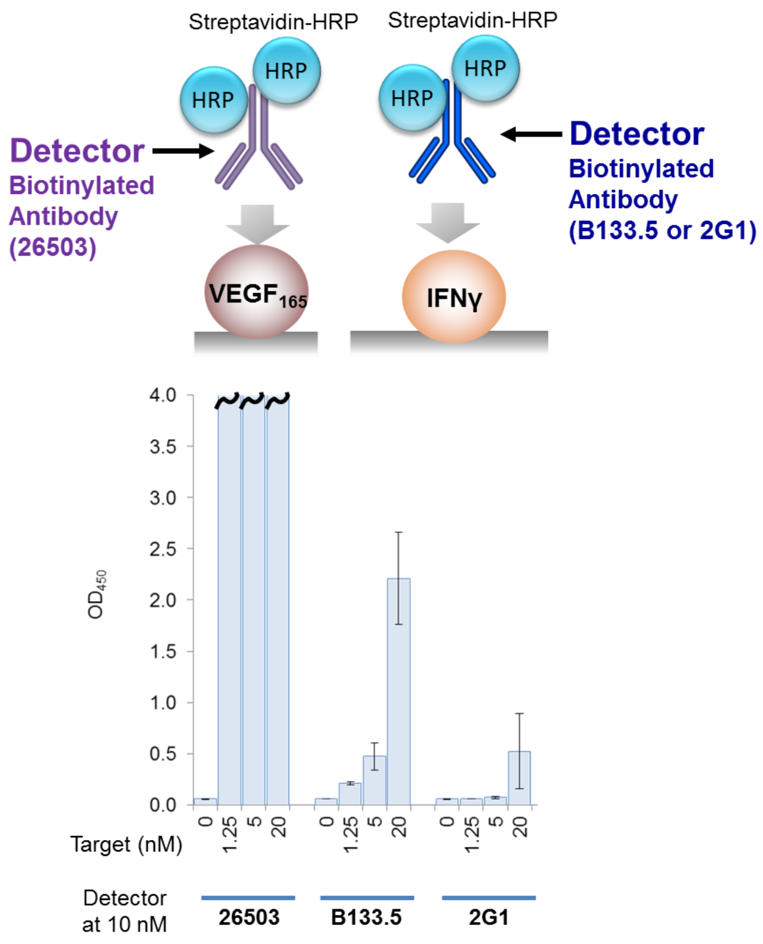


**Supplementary Figure S3. Detection of the target proteins using monoclonal antibodies as the primary detector agents**. The target was directly immobilized on the plate surface by an incubation with the target solution (100 µl per well) at each indicated concentration, in the presence of 1 µg/ml of BSA in 0.1 M carbonate buffer (pH 9.6), for 2 hours. After the incubation, the plate surfaces were further coated with blocking solution (1% BSA in 1× D-PBS(-), 300 µl per well) for 2 hours. After the blocking reaction, 100 µl of the detector solution (10 nM biotinylated each monoclonal antibody in 1× binding buffer) was added to each well and then the binding reaction was performed for 30 min. After the incubation, 100 µl of the secondary detector solution (50 ng/ml HRP-conjugated streptavidin in 1× binding buffer) was added to each well, and then incubated for 30 min. After washing the wells, the TMB reaction (100 µl per well) was performed for 5 min ($VEGF_{165}$) or 15 min (IFNγ). The sample size is two per each combination set, and the error bars represent one standard deviation. The bars with wavy lines indicate that at least one of the two sample wells showed overflow (OD450 > 4.000).

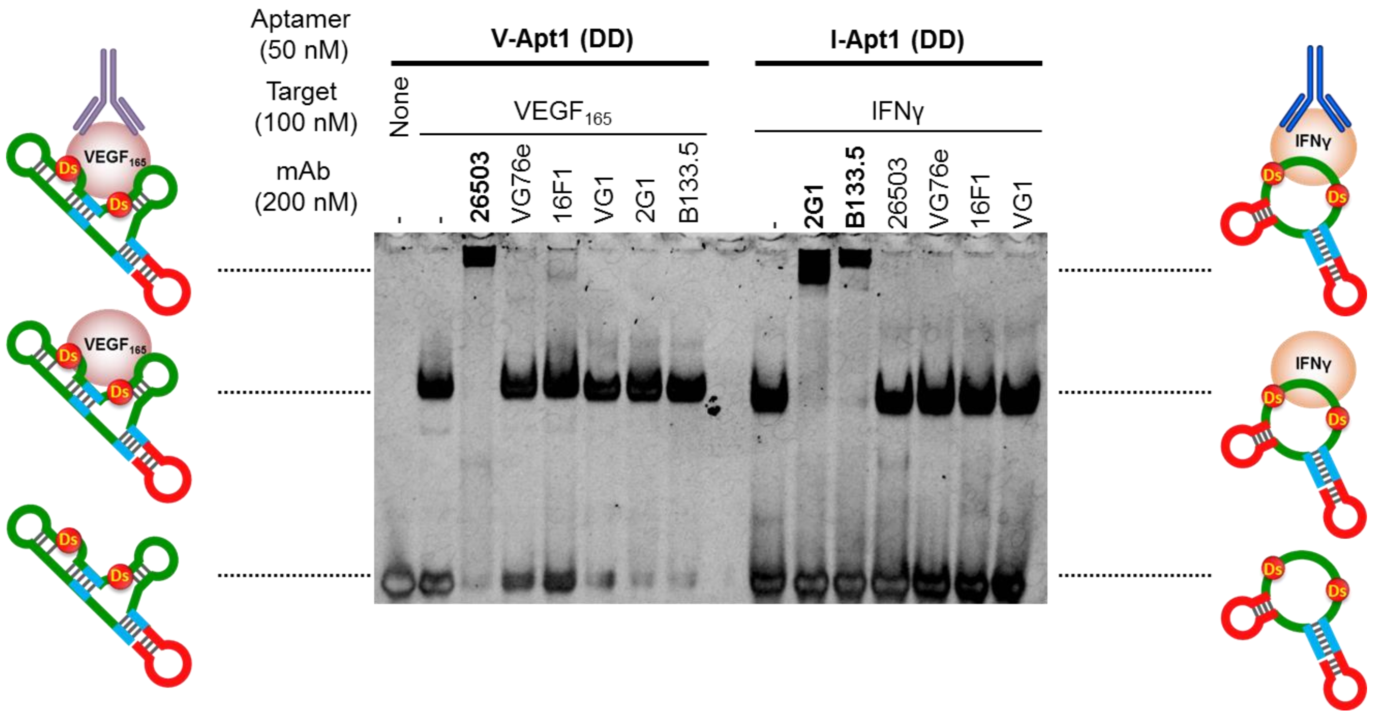


**Supplementary Figure S4. Gel shift assay to find sandwich aptamer-antibody pairs for target detection.** A gel shift assay was used to screen for super-complex formation among three components: aptamer, its target, and monoclonal antibody (mAb). The anti-$VEGF_{165}$ mAbs are 26503, VG76e, 16F1, and VG1 and the anti-IFNγ mAbs are 2G1 and B133.5. For the binding reactions, 10 µl of DNA aptamer solution (100 nM, in 1× PBS supplemented with 10% glycerol) was mixed with 5 µl of the target solution (400 nM of $VEGF_{165}$ or IFNγ in 1× PBS supplemented with 0.05% Nonidet P-40) and 5 µl of each mAb solution (0 or 800 nM mAb in 1× PBS supplemented with 0.05% Nonidet P-40). After an incubation at 25°C for 15 min, 15 µl of the mixture solution was loaded onto a 6% native polyacrylamide gel (0.5 ×TBE with 5% glycerol), and electrophoresed at room temperature. The DNA bands on the gel were detected by staining with SYBR Gold, and the band patterns were obtained with an LAS-4000 imager (Fuji Film). The super-shifted band patterns clearly showed that the anti-$VEGF_{165}$ mAb, 26503, and the anti-IFNγ mAbs, 2G1 and B133.5, can bind to their cognate aptamer-target binary complexes.

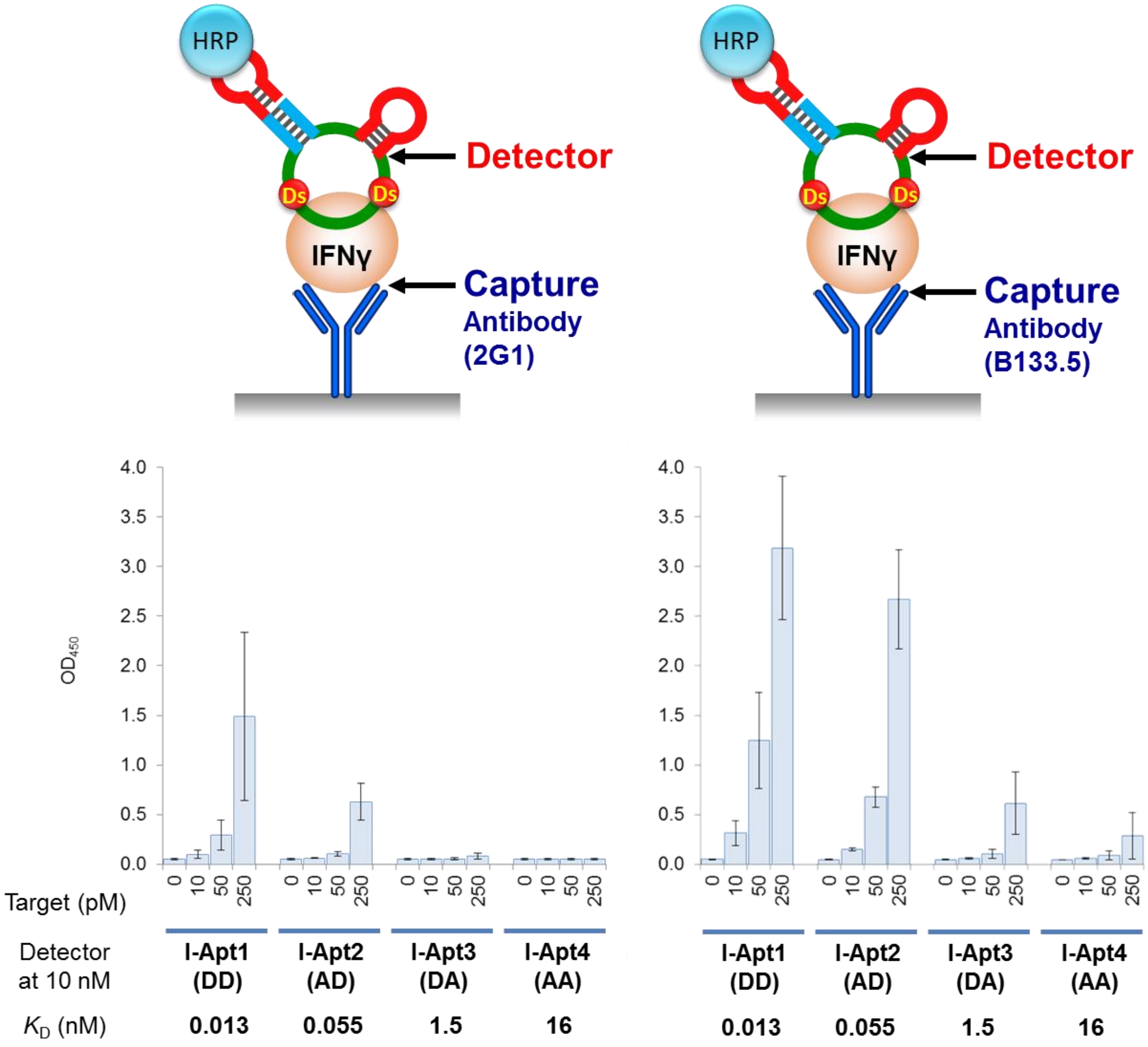


**Supplementary Figure S5. Sandwich-type detection by each anti-IFNγ aptamer variant as the primary detector agent in the combination of the pairing monoclonal antibody (2G1 or B133.5) as the capture agent.** The target protein (IFNγ) was indirectly immobilized (captured) on the plate surface via each corresponding monoclonal antibody, by an incubation with the target solution (100 µl per well, in 1× binding buffer) at each indicated concentration. After the indirect immobilization, 100 µl of the primary detector solution (10 nM each aptamer variant in 1× binding buffer) was added to each well and then the binding reaction was performed for 30 min. After the incubation, 100 µl of the secondary detector solution (50 ng/ml HRP-conjugated streptavidin in 1× binding buffer) was added to each well, followed by an incubation for 30 min. After washing the wells, the TMB reaction (100 µl per well) was performed for 15 min. The sample size is two per each combination set, and the error bars represent one standard deviation. The results show that the mAb with higher affinity is better as a capture agent, and the aptamer variant with higher affinity is better as a detector agent.

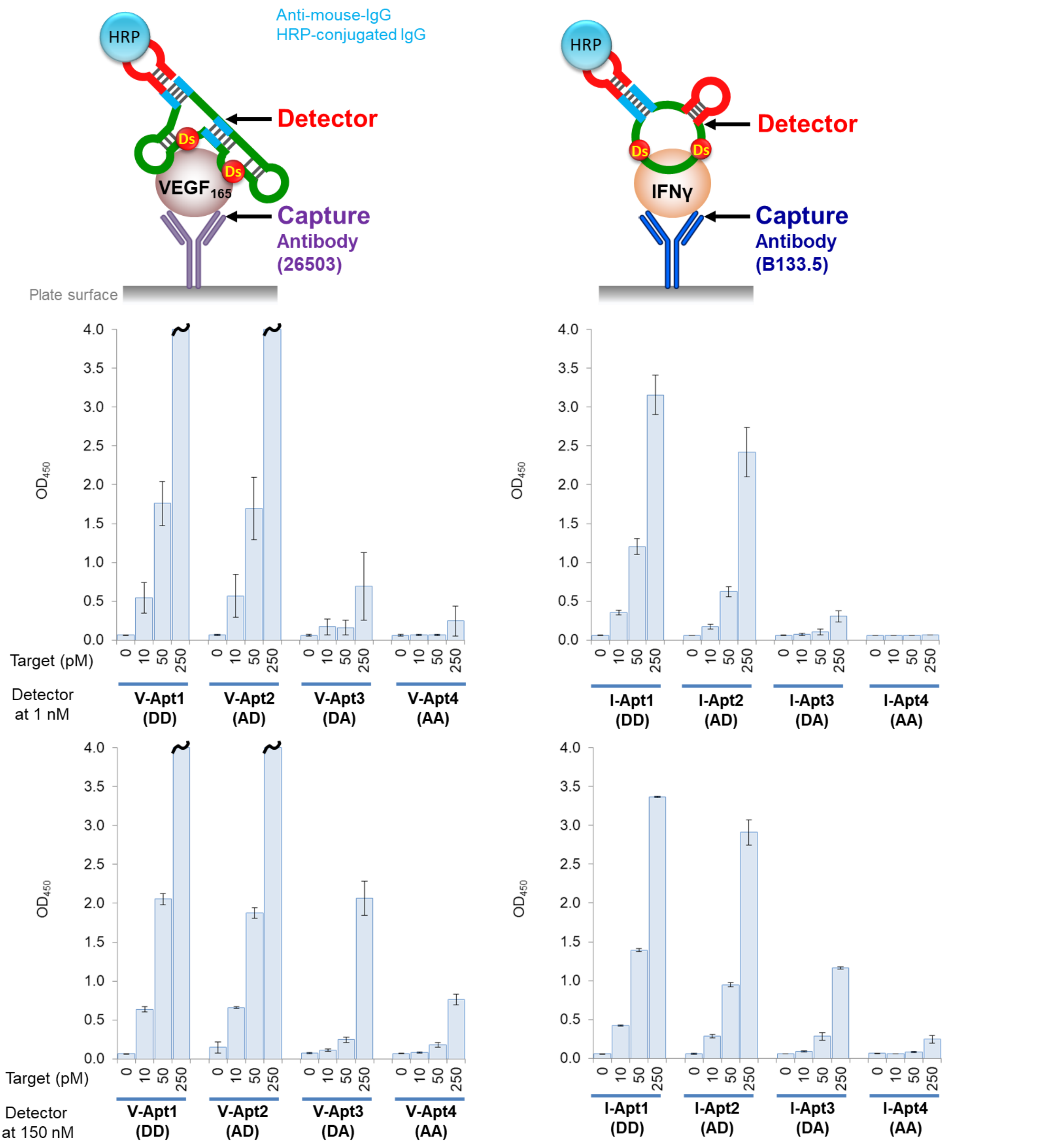


**Supplementary Figure S6. Sandwich-type detection by each aptamer variant as a detector agent in the combination of the pairing monoclonal antibody (mAb) as the capture agent.** The target was indirectly immobilized on the plate surface via each corresponding mAb as the capture agent, by an incubation with the target solution (100 µl per well, in 1× binding buffer) at each indicated concentration. After the indirect immobilization, 100 µl of the detector solution (1 nM or 150 nM each aptamer variant in 1× binding buffer) was added to each well and the binding reaction was performed for 30 min. After the incubation and washing, 100 µl of the secondary detector solution (50 ng/ml HRP-conjugated streptavidin in 1× binding buffer) was added to each well, followed by an incubation for 30 min. After washing the wells, the TMB reaction (100 µl per well) was performed for 15 min. The sample sizes are four (at 1 nM) and two (at 150 nM) per each combination set, and the error bars represent one standard deviation. The bars with wavy lines indicate that at least one of the two sample wells showed overflow ($OD_{450}$ > 4.000).

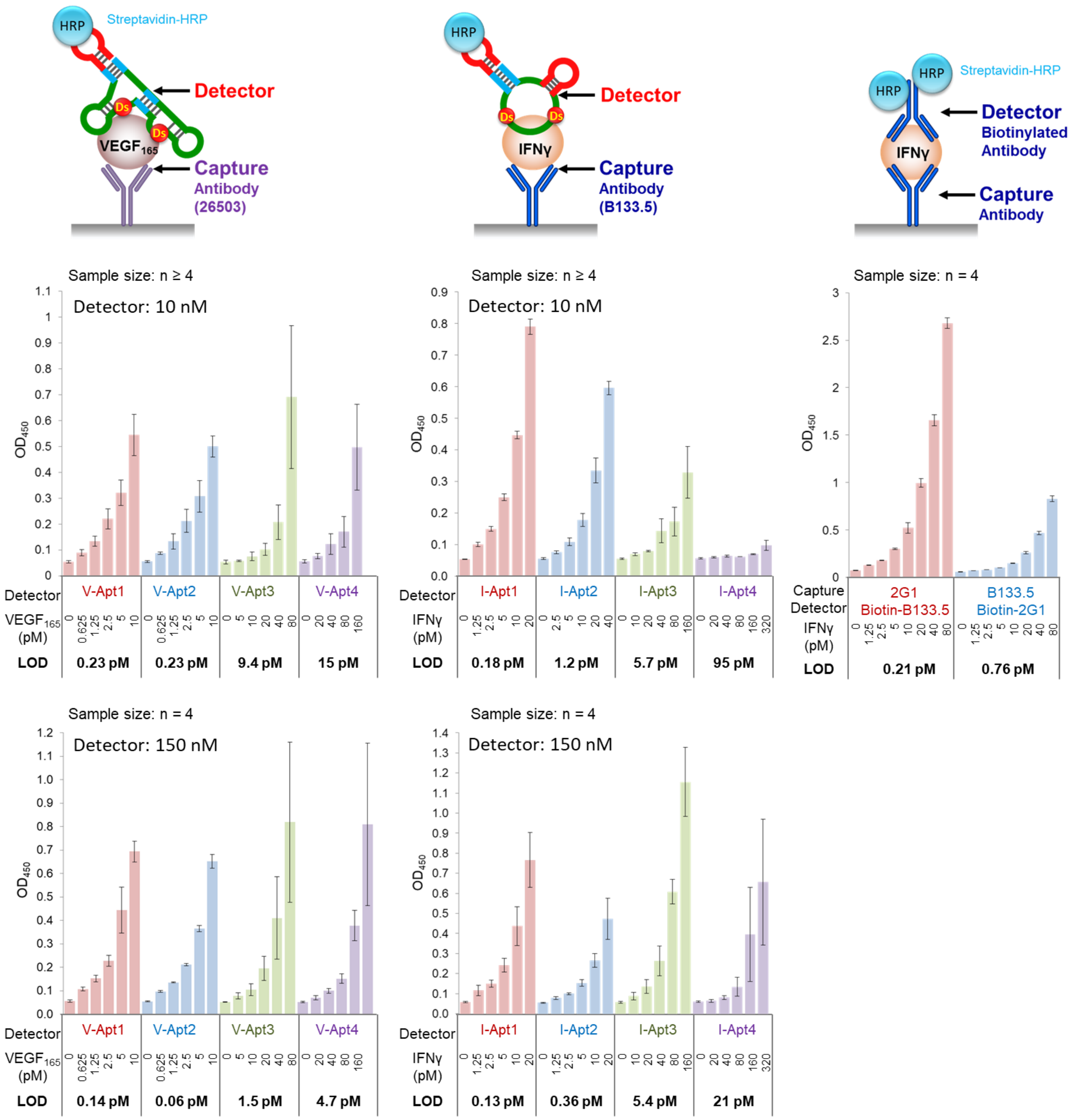


**Supplementary Figure S7. Determination of limit of detection (LODs) targeting VEGF$_{165}$ and IFNγ by a sandwich-type ELISA, using antibodies as capture agents.** The sample size is four or more in each combination set. The error bars represent one standard deviation.

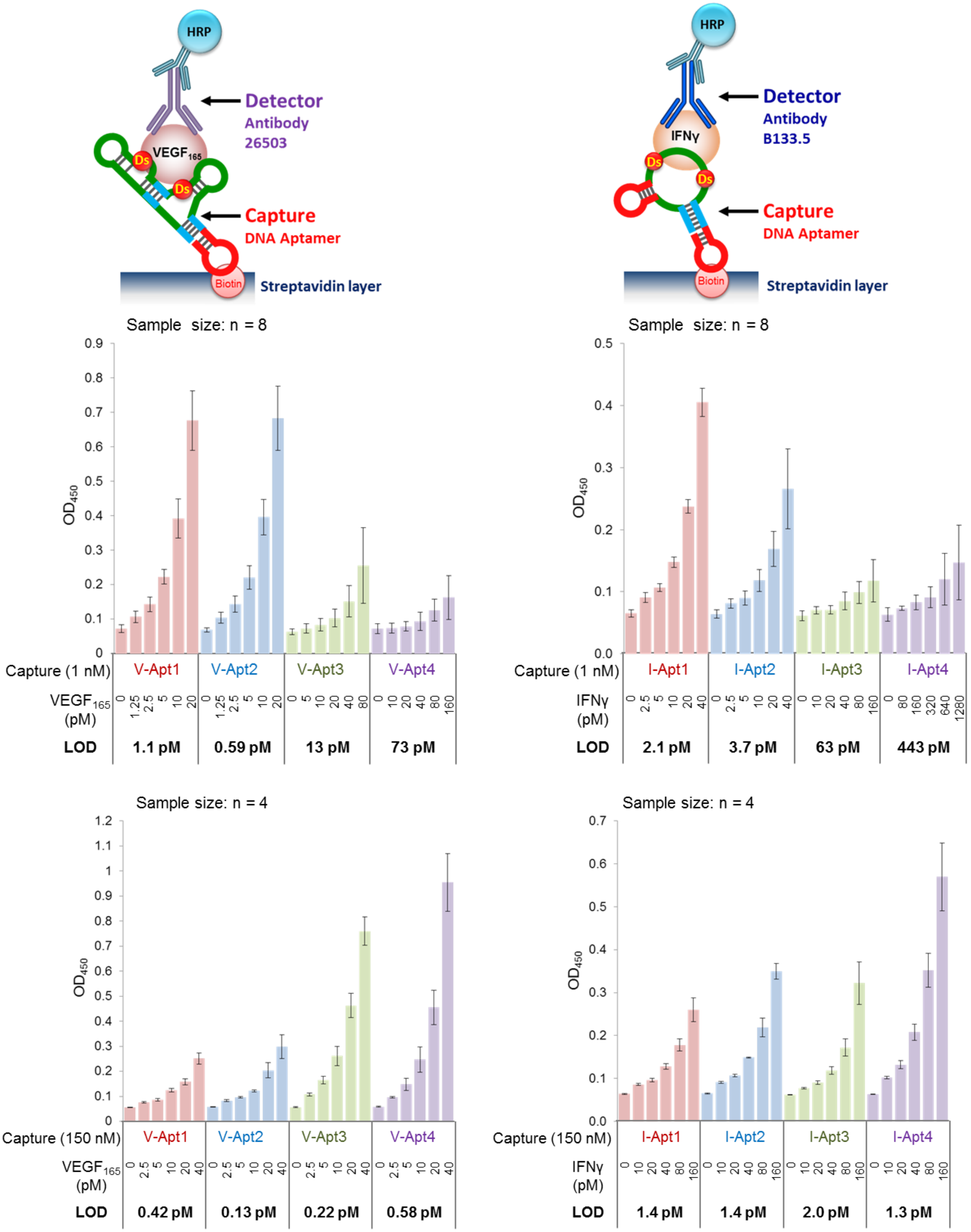


**Supplementary Figure S8. Determination of limit of detection (LODs) targeting $VEGF_{165}$ and IFNγ by a sandwich-type ELISA, using aptamers as capture agents on the plates coated with streptavidin.**

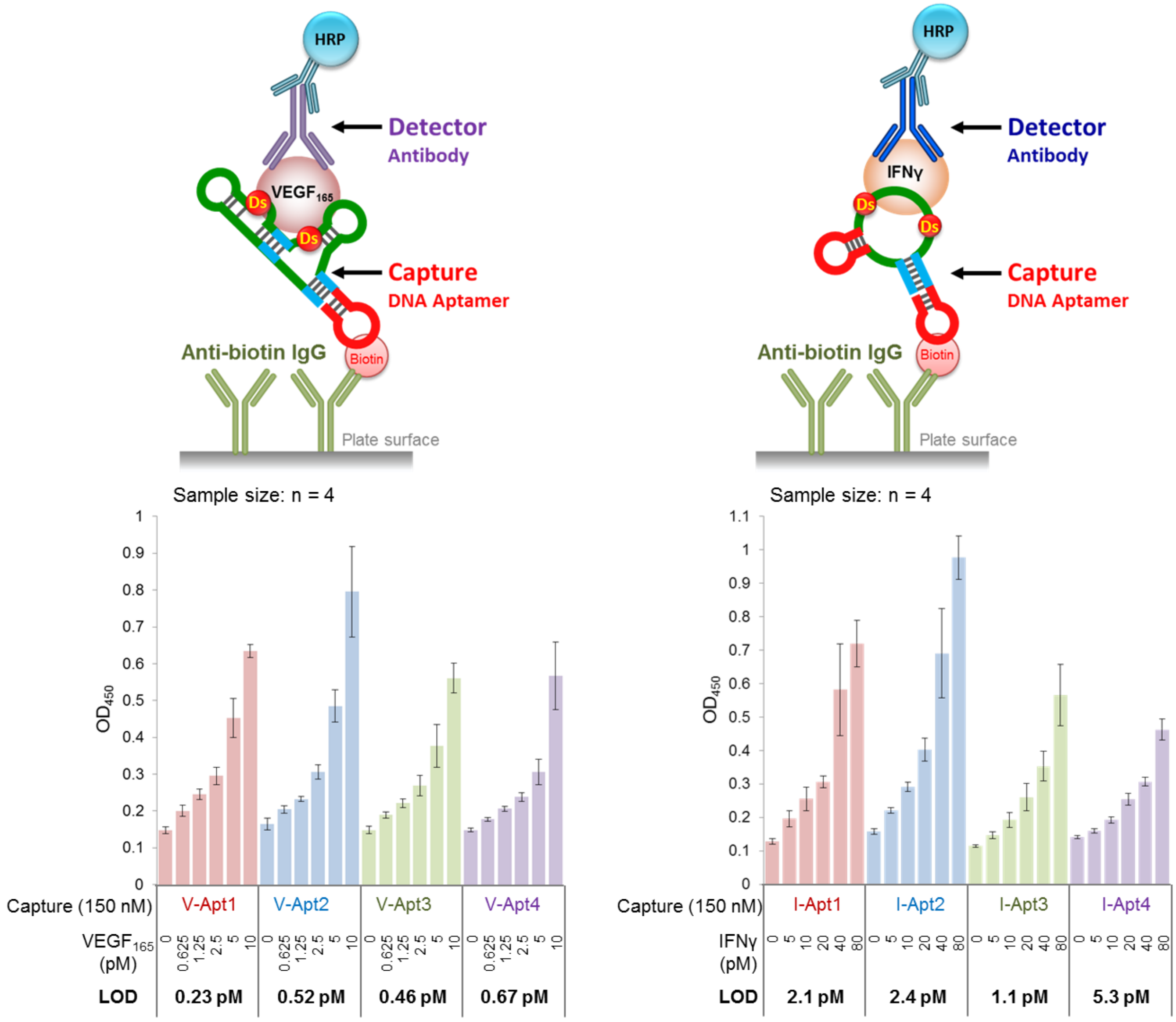


**Supplementary Figure S9. Determination of limit of detection (LODs) targeting $VEGF_{165}$ and IFNγ by a sandwich-type ELISA, using aptamers as capture agents on the plates coated with anti-biotin IgG.**

| Target | Plate Pre-coating | Capture Agent | Capture Conc. (nM) | Primary Detector Agent | Primary Detector Conc. (nM) | LOD (pM) |
|---|---|---|---|---|---|---|
| $VEGF_{165}$ | None | 26503 | 20 | V-Apt1 | 10 | 0.23 |
| | | | | V-Apt2 | | 0.23 |
| | | | | V-Apt3 | | 9.7 |
| | | | | V-Apt4 | | 15 |
| | None | 26503 | 20 | V-Apt1 | 150 | 0.14 |
| | | | | V-Apt2 | | 0.06 |
| | | | | V-Apt3 | | 1.5 |
| | | | | V-Apt4 | | 4.7 |
| | Streptavidin | V-Apt1 | 1 | 26503 | 10 | 1.1 |
| | | V-Apt2 | | | | 0.59 |
| | | V-Apt3 | | | | 13 |
| | | V-Apt4 | | | | 73 |
| | Streptavidin | V-Apt1 | 150 | | | 0.42 |
| | | V-Apt2 | | | | 0.13 |
| | | V-Apt3 | | | | 0.22 |
| | | V-Apt4 | | | | 0.58 |
| | Anti-biotin IgG | V-Apt1 | 150 | | | 0.23 |
| | | V-Apt2 | | | | 0.52 |
| | | V-Apt3 | | | | 0.46 |
| | | V-Apt4 | | | | 0.67 |

| Target | Plate Pre-coating | Capture Agent | Capture Conc. (nM) | Primary Detector Agent | Primary Detector Conc. (nM) | LOD (pM) |
|---|---|---|---|---|---|---|
| IFNγ | None | B133.5 | 20 | I-Apt1 | 10 | 0.18 |
| | | | | I-Apt2 | | 1.2 |
| | | | | I-Apt3 | | 5.7 |
| | | | | I-Apt4 | | 95 |
| | None | B133.5 | 20 | I-Apt1 | 150 | 0.13 |
| | | | | I-Apt2 | | 0.36 |
| | | | | I-Apt3 | | 5.4 |
| | | | | I-Apt4 | | 21 |
| | Streptavidin | I-Apt1 | 1 | B133.5 | 10 | 2.1 |
| | | I-Apt2 | | | | 3.7 |
| | | I-Apt3 | | | | 63 |
| | | I-Apt4 | | | | 443 |
| | Streptavidin | I-Apt1 | 150 | | | 1.4 |
| | | I-Apt2 | | | | 1.4 |
| | | I-Apt3 | | | | 2 |
| | | I-Apt4 | | | | 1.3 |
| | Anti-biotin IgG | I-Apt1 | 150 | | | 2.1 |
| | | I-Apt2 | | | | 2.4 |
| | | I-Apt3 | | | | 1.1 |
| | | I-Apt4 | | | | 5.3 |
| | None | B133.5 | 20 | 2G1 | 10 | 0.76 |
| | | 2G1 | 20 | B133.5 | 10 | 0.21 |

| |
|---|
| 0.05 |
| 1 |
| 2 |
| 100 |
| 500 |

**Supplementary Figure S10. Summary of the limit of detections (LODs) targeting $VEGF_{165}$ and IFNγ by a sandwich-type ELISA, using Ds-DNA aptamers and their variants as detector or capture agents in combination with each cognate antibody.** For comparison, the antibody-antibody combination patterns are also included.

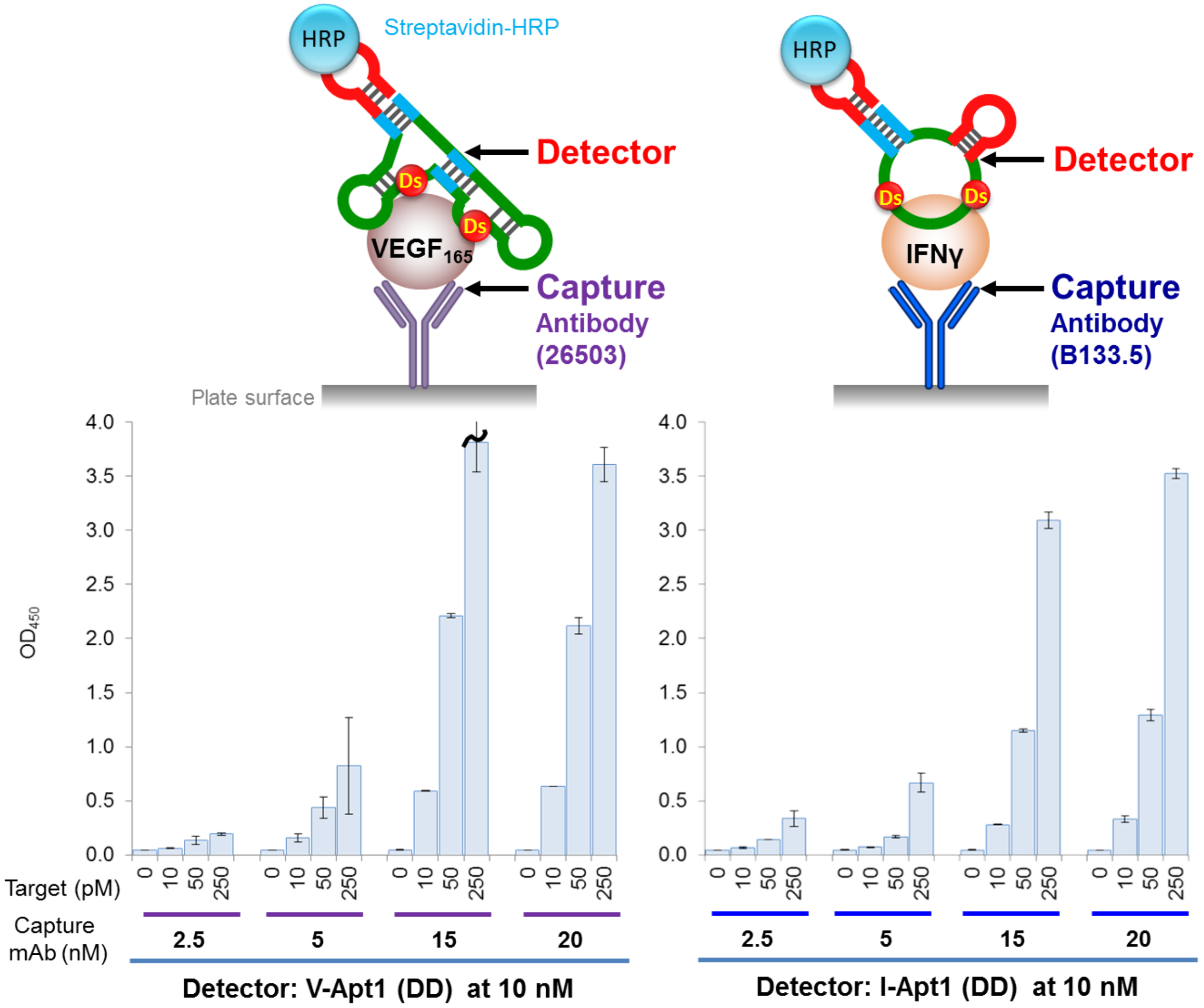


**Supplementary Figure S11. Concentration dependency of monoclonal antibodies as capture agents in the sandwich-type detection.** Different concentrations of the antibody (2.5 nM to 20 nM in 0.1 M carbonate buffer (pH 9.6), 50 µl per well) were immobilized on the plate surface, by an incubation for 2 hours, followed by blocking with BSA. The target binding was then performed by an incubation with the target solution (100 µl per well, in 1× binding buffer) at each indicated concentration. After the antibody-target binding, 100 µl of the primary detector solution (10 nM DNA aptamer, V-Apt1 (DD) or I-Apt1 (DD)) was added to each well and the plate was incubated for 30 min. After the incubation, 100 µl of the secondary detector solution (50 ng/ml HRP-conjugated streptavidin in 1× binding buffer) was added to each well, followed by an incubation for 30 min. After washing the wells, the TMB reaction (100 µl per well) was performed for 15 min. The sample size is two per each combination set, and the error bars represent one standard deviation. The results show that the target detection sensitivity became lower when the amount of capture agent decreased.

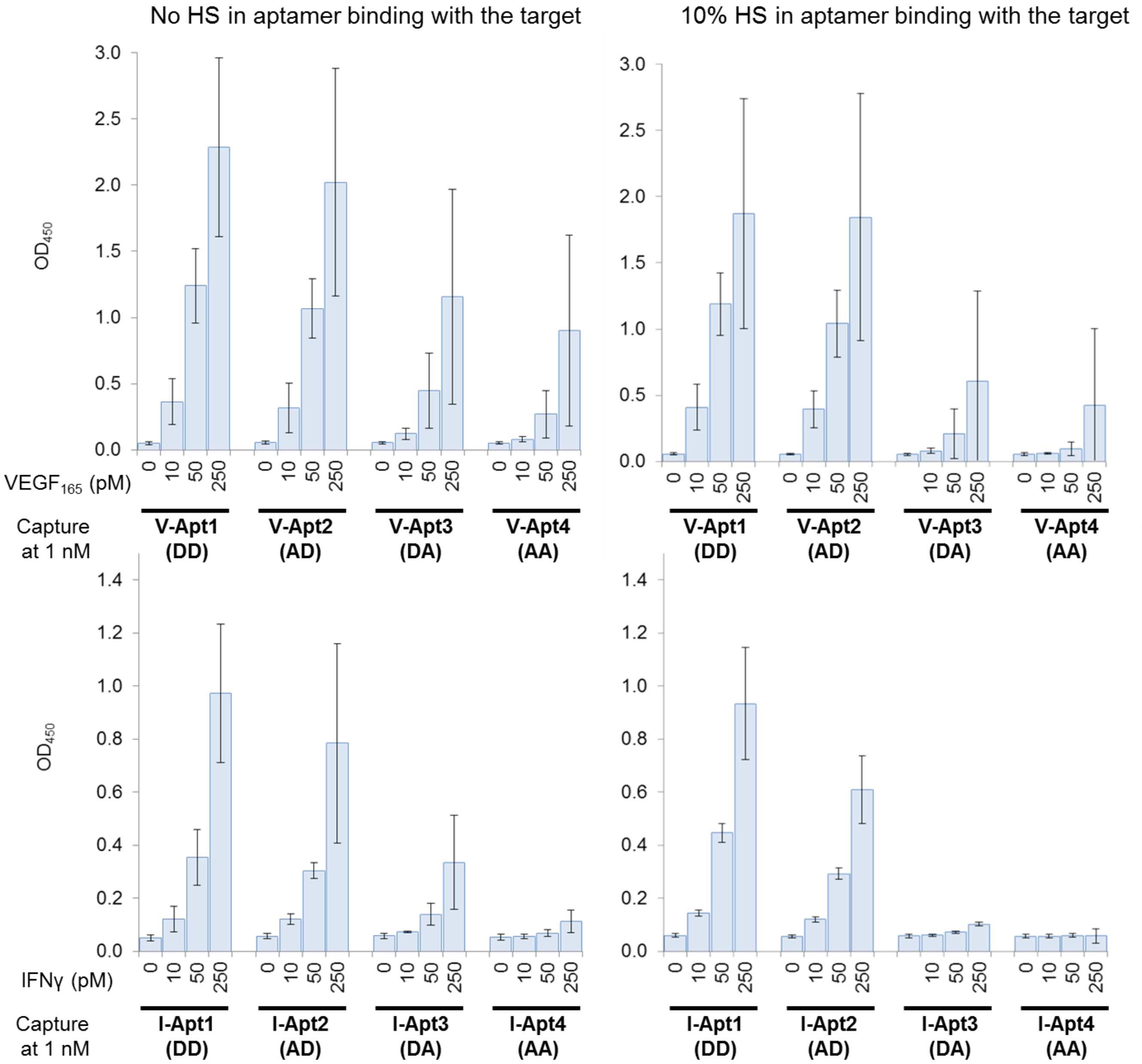


**Supplementary Figure S12. Sandwich-type detection by each aptamer variant as a capture agent in the presence of human serum (HS).** The aptamer was immobilized on the plate surface coated with streptavidin, by an incubation for 30 min at 1 nM in 1 × binding buffer (100 µl per well). The target binding was then performed by an incubation with the target solution (100 µl per well, in 1× binding buffer or 10% HS / 0.9× binding buffer) at each indicated concentration. After the aptamer-target binding, 100 µl of the detector solution (10 nM each monoclonal antibody in 1× binding buffer) was added to each well and the plate was incubated for 30 min. After the incubation, 100 µl of the secondary detector solution (50 ng/ml HRP-conjugated anti-mouse IgG antibody in 1× binding buffer) was added to each well, followed by an incubation for 30 min. After washing the wells, the TMB reaction (100 µl per well) was performed for 15 min. The data in the absence of HS are the same as those shown in Figure 6. The sample size is n = 6 in the presence of HS, and the error bars represent one standard deviation. The results show that the target detection sensitivity by the high-affinity aptamers was not largely affected in the presence of HS, indicating that their binding was not inhibited by HS.

**Supplementary Table S1. DNA sequences used in this study.** Sequences of the original anti-VGEF$_{165}$ DNA aptamer (58-mer, V-Apt1) and anti-IFNγ DNA aptamer (57-mer, I-Apt1), as well as their variants, are listed. The positions of the hydrophobic Ds bases (indicated as "I" in red) and biotinylated T (indicated as "L" in blue) are shown in bold. The mini-hairpin DNA sequences are underlined.

| **Sample** | **Sequence (5' to 3')** **I** = Ds, **L** = Biotin-dT |
|---|---|
| V-Apt1 (DD) | GCGGTAAGCCGCGTCCGAAGGGGC**I**TGCGGCGA**I**CCCGAATGGGTCCGCCGCG**L**AGCG |
| V-Apt2 (AD) | GCGGTAAGCCGCGTCCGAAGGGGC**A**TGCGGCGA**I**CCCGAATGGGTCCGCCGCG**L**AGCG |
| V-Apt3 (DA) | GCGGTAAGCCGCGTCCGAAGGGGC**I**TGCGGCGA**A**CCCGAATGGGTCCGCCGCG**L**AGCG |
| V-Apt4 (AA) | GCGGTAAGCCGCGTCCGAAGGGGC**A**TGCGGCGA**A**CCCGAATGGGTCCGCCGCG**L**AGCG |
| I-Apt1 (DD) | CCCGCCCGGGTCCGCGAAGCGGTAGGT**I**TGGGCTAGGC**I**GCTGGCGGGCGCG**L**AGCG |
| I-Apt2 (AD) | CCCGCCCGGGTCCGCGAAGCGGTAGGTATGGGCTAGGC**I**GCTGGCGGGCGCG**L**AGCG |
| I-Apt3 (DA) | CCCGCCCGGGTCCGCGAAGCGGTAGGT**I**TGGGCTAGGC**A**GCTGGCGGGCGCG**L**AGCG |
| I-Apt4 (AA) | CCCGCCCGGGTCCGCGAAGCGGTAGGT**A**TGGGCTAGGC**A**GCTGGCGGGCGCG**L**AGCG |
