# Supplementary material for: Molecular affinity rulers: systematic evaluation of DNA aptamers for their applicabilities in ELISA
Source: Nucleic Acids Res. 2019 Aug 8;47(16):8362–74. doi: 10.1093/nar/gkz688 (PMC6895277; doi:10.1093/nar/gkz688)
Supplement: gkz688_Supplemental_Files [file gkz688_supplemental_files.zip › Replacement - July 30 - gkz688 - Supplemental Material.pdf]

Supplemental Material

A 212-nt long RNA structure in the Tobacco necrosis virus-D RNA genome is resistant to Xrn degradation

Chaminda D. Gunawardene, Laura R. Newburn and K. Andrew White

In vitro Xrn1 assays of sg mRNA1 mutants

For all Figures, the top panel shows a northern blot of the untreated wt and mutant sg mRNA1s. The middle panels shows a northern blot of the svRNAs resulting from Xrn1 treatment of the sg mRNA1s shown in the upper panel. Values for svRNA levels were derived from quantification of the northern blots (level of svRNA to level of corresponding untreated sg mRNA). The lower panel shows ethidium bromide (EtBr) stained gels of svRNAs in the middle panel before they were transferred to nylon and probed. BPB, bromophenol blue.

Representative Northern Blots and EtBr stained gels of sg mRNA1 and svRNAs used for quantification of values presented in **Figure 3D**

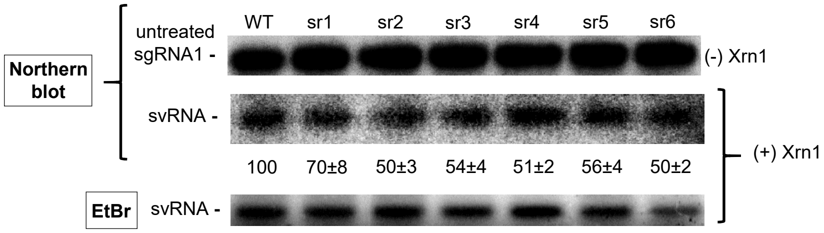

Fig S1. Mutants sr1 through sr6

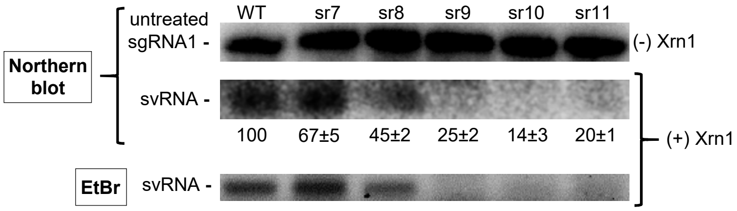

Fig S2: Mutants sr7 through sr11

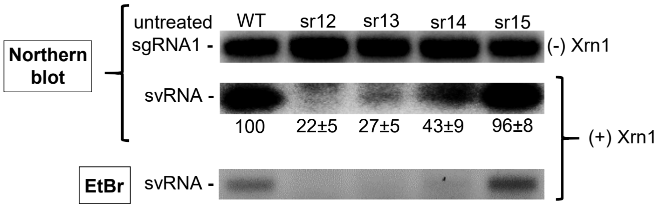

Fig S3: Mutants sr12 through sr15

Representative Northern Blots and EtBr stained gels of sg mRNA1 and svRNAs used for quantification of values presented in **<u>Figure 4A</u>** for mutations in the **<u>3'-CITE</u>**

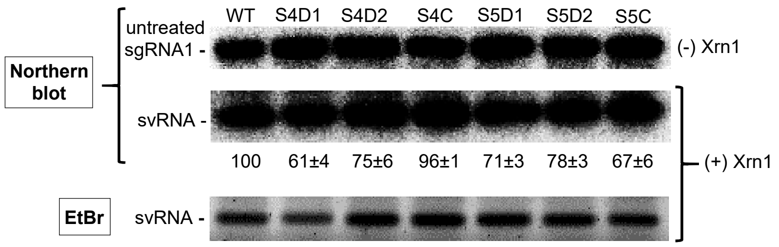

Fig S4: Mutant series S4 and S5

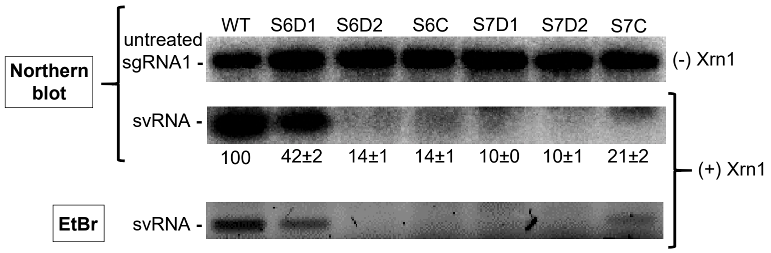

Fig S5: Mutant series S6 and S7

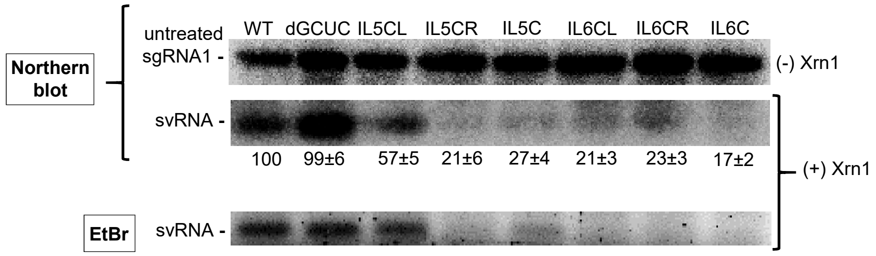

Fig S6: Mutant series IL5 and IL6, and mutant dGCUC

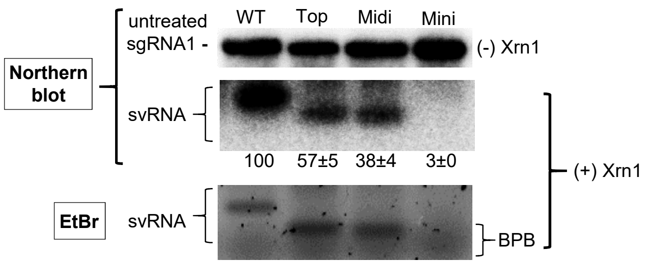

Fig S7: Mutants Top, Midi, and Mini
(**<u>Note</u>:** non-correlating EtBr staining is due to size/structure differences)

Representative Northern Blots and EtBr stained gels of sg mRNA1 and svRNAs used for quantification of values presented in **<u>Figure 4A</u>** for mutations in **<u>SLII</u>**

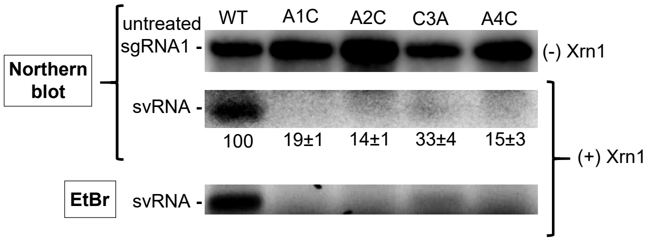

Fig S8: SLII – Terminal Loop mutants

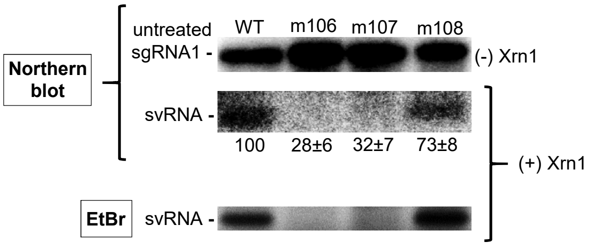

Fig S12: SLII – Lower stem mutants

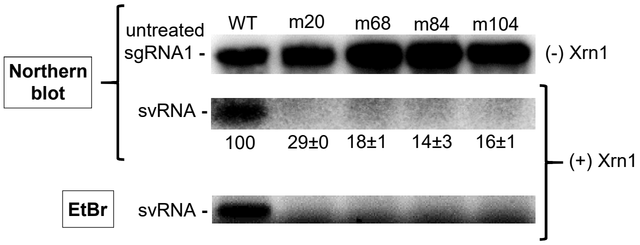

Fig S9: SLII – Terminal Loop mutants

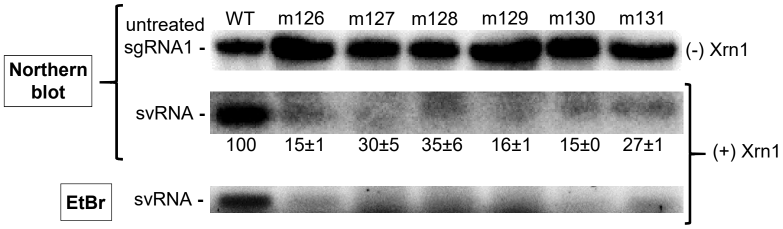

Fig S10: SLII – Upper stem mutants

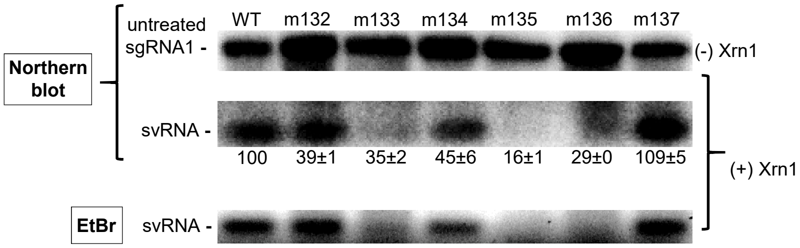

Fig S11: SLII – Upper stem mutants
